# Inhibition of Wnt/*β*-Catenin pathway and Histone acetyltransferase activity by Rimonabant: a therapeutic target for colon cancer

**DOI:** 10.1038/s41598-017-11688-x

**Published:** 2017-09-15

**Authors:** Maria Chiara Proto, Donatella Fiore, Chiara Piscopo, Silvia Franceschelli, Valentina Bizzarro, Chiara Laezza, Gianluigi Lauro, Alessandra Feoli, Alessandra Tosco, Giuseppe Bifulco, Gianluca Sbardella, Maurizio Bifulco, Patrizia Gazzerro

**Affiliations:** 1 Department of Pharmacy, University of Salerno, Fisciano, 84084 Italy; 2 Institute of Endocrinology and Experimental Oncology, IEOS CNR, Naples, 80131 Italy; 3 Department of Medicine, Surgery and Dentistry “Scuola Medica Salernitana”, University of Salerno, Baronissi, 84081 Italy

## Abstract

In a high percentage (≥85%) of both sporadic and familial adenomatous polyposis forms of colorectal cancer (CRC), the inactivation of the APC tumor suppressor gene initiates tumor formation and modulates the Wnt/*β*-Catenin transduction pathways involved in the control of cell proliferation, adhesion and metastasis. Increasing evidence showed that the endocannabinoids control tumor growth and progression, both *in vitro* and *in vivo*. We evaluated the effect of Rimonabant, a Cannabinoid Receptor 1 (CB1) inverse agonist, on the Wnt/*β*-Catenin pathway in HCT116 and SW48 cell lines carrying the genetic profile of metastatic CRC poorly responsive to chemotherapies. In these models, Rimonabant inhibited the Wnt/*β*-Catenin canonical pathway and increased *β*-Catenin phosphorylation; in HCT116 cells, but not in SW48, the compound also triggered the Wnt/*β*-Catenin non canonical pathway activation through induction of Wnt5A and activation of CaMKII. The Rimonabant-induced downregulation of Wnt/*β*-Catenin target genes was partially ascribable to a direct inhibition of p300/KAT3B histone acetyltransferase, a coactivator of *β*-Catenin dependent gene regulation. Finally, in HCT116 xenografts, Rimonabant significantly reduced tumor growth and destabilized the nuclear localization of *β*-Catenin. Obtained data heavily supported the rationale for the use of cannabinoids in combined therapies for metastatic CRC harbouring activating mutations of *β*-Catenin.

## Introduction

The majority of both sporadic and familial forms of adenomatous polyposis (FAP) in colorectal cancer (CRC) originates from inactivation of APC (Adenomatous Polyposis Coli) tumor suppressor gene. APC negatively regulates the levels of *β*-Catenin that transduces Wnt signals, mediates cell-cell adherent junctions through its interaction with E-cadherin, and stimulates cell proliferation. WNTs are able to modulate both the ‘canonical’ *β*-Catenin-dependent and the ‘non-canonical’ *β*-Catenin-independent Wnt signalling pathways^1^. In the canonical pathway, in the absence of Wnt ligands, GSK3 (Glycogen Synthase Kinase 3), casein kinase 1 *α* (CK1 *α*), axin and APC promote the phosphorylation of *β*-Catenin, its ubiquitylation and degradation. Interaction of Wnt ligands, such as Wnt3a, with Frizzled (Fzd) receptors and the Wnt co-receptor low density Lipoprotein Receptor-related Protein 5 (LRP5) or LRP6 activates the Dishevelled (Dvl) cytoplasmic phospho proteins, which inhibit *β*-Catenin phosphorylation and block its degradation. Then *β*-Catenin accumulates in the nucleus, binds to Lymphoid Enhancer-binding Factor (LEF) and T Cell Factor (TCF) proteins and acts as a transcriptional co-activator to modulate the expression of target genes^2^. Some WNTs, such as Wnt5A and Wnt11, fail to stabilize *β*-Catenin and can also induce a calcium flux and the activation of various pathways, such as PKC (Protein kinase C), CaMKII (Calcium/Calmodulin Dependent Protein Kinase II) and JNK (c-Jun N-terminal kinases)^3^. Moreover, Wnt5A inhibits Wnt3A-induced canonical pathway in a dose-dependent manner^4^. Wnt5A is frequently silenced in human CRC cell lines and in human primary tumors due to its promoter methylation, resulting thus as a potential epigenetic biomarker or therapeutic target for CRC^5^. Wnt/*β*-Catenin signalling regulates different cellular processes in both embryonic and adult stages and increasing evidence suggests that Wnt target genes are mainly cell and context specific. The multifaceted role of Wnt/*β*-Catenin pathway is a topic still debated and the analysis of the Wnt targetome^6^ and of *β*-Catenin target genes^7^ identified new players associated with CRC. Intriguingly, anandamide (AEA), a cannabinoid receptor 1 (CB1) agonist, inhibits cholangiocarcinoma growth through activation of the non-canonical Wnt pathway mediated by Wnt5A^8^ and, in human breast cancer cells, methyl-F-AEA reduces *β*-Catenin levels, inhibits the transcriptional activation of TCF responsive elements and decreases the expression of mesenchymal markers^9^. These data strongly suggest our hypothesis of a potential effect of cannabinoids on the Wnt/*β*-Catenin in CRC, but to date no data dissected these interactions. The endocannabinoid (EC) system possesses antitumor effects *in vitro* and *in vivo* ^10–12^. Δ9-tetrahydrocannabinol induces apoptosis in CRC by CB1-mediated inhibition of both RAS-MAPK/ERK and PI3K-Akt signalling and activation of BAD (BCL2 Associated Agonist of cell Death)^10^. In DLD1 and HT29 CRC cell lines, both CB1 and CB2 (cannabinoid receptor 2) receptor agonists induce apoptosis^13^. In the azoxymethane (AOM) induced aberrant crypt foci (ACF) model, the inhibition of EC hydrolysis decreases the development of precancerous lesions in the mouse colon^14^. Moreover, cannabinoids significantly reduce the proliferation of CRC cell lines^15^ and raise the expression of CB1 and estrogen receptors (ER)^15^, whose loss might promote and accelerate colorectal carcinogenesis in APC^Min/+^ mice^16^. Last but not least, cannabinoids improve the efficacy of chemotherapic drugs used in the clinical practice^17, 18^. A priority in the treatment of human cancer is the finding of strategies able to reduce the occurrence of resistance to chemotherapies or, alternatively, to identify new pathways as targets of drugs enabling to bypass these events. In this issue the ECs could represent good therapeutic chances acting at several levels. We previously found that among other cannabinoid compounds, Rimonabant (SR141716), an inverse agonist at the CB1 receptor, shows a powerful antitumor effect both in precancerous lesions and in CRC cell lines^11, 17, 18^. Here we dissected the role of Rimonabant in CRC analyzing, *in vitro* and *in vivo*, its effects on the Wnt/*β*-Catenin mediated signalling.

## Results

### SR141716 inhibits proliferation in human CRC cells *in vitro*

**Figure 1:**
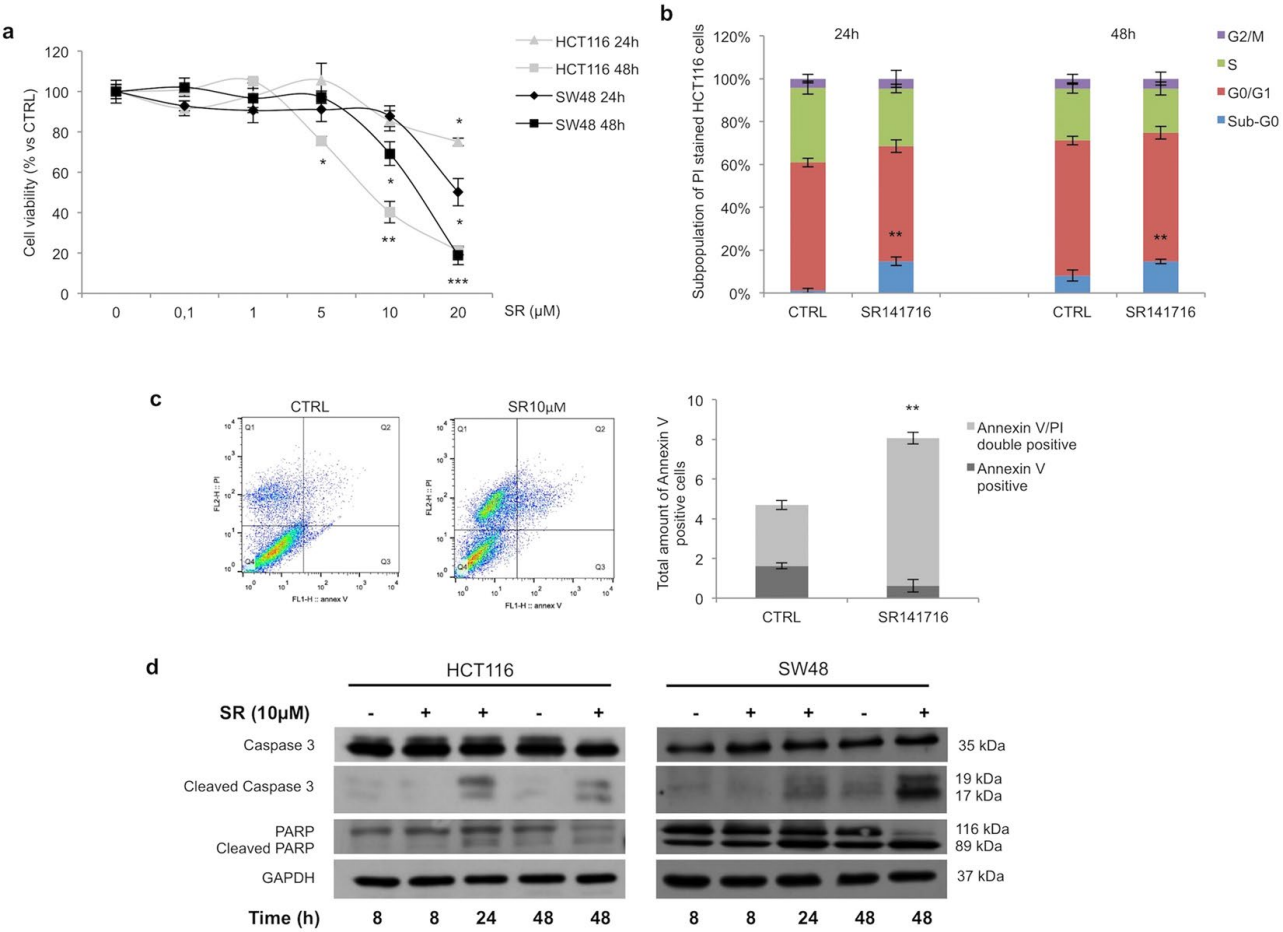
Effect of SR141716 on cell viability. Cell viability (**a**), cell cycle analysis using propidium iodide staining (**b**) and apoptosis analysis (**c**) using AnnexinV-FITC/PI of HCT116 cells treated with SR141716 or vehicle alone. (**d**) Representative western blot analysis of Caspase 3 and PARP (total and cleaved forms) expression in total protein lysates from HCT116 and SW48 cells untreated (−) or treated (+) with the compound (mean ± SD; unpaired two tailed Student’s t-test *p < 0.05, **p < 0.01 and ***p < 0.005). Cropped blots from full-length gels are displayed in d.

We previously found that in CRC cell lines, DLD1 and SW620, SR141716 induced G2/M and S-G2/M arrest, respectively, without induction of apoptosis or necrosis^11^. Here we found that SR141716 inhibited HCT116 cell growth (Fig. 1a), induced a significant increase of Sub-G0/G1 cell phase, persistent until 48 hours (Fig. 1b) and raised the percentage of PI/Annexin V-FITC double stained cells (Fig. 1c). In both HCT116 and SW48 cell lines, the increase of Caspase 3- and PARP-cleaved protein levels starting from 24 hours (Fig. 1d and see Supplementary Fig. S1a) strongly suggested induction of apoptosis. This was also confirmed by results from human apoptosis antibody array, performed in HCT116 cells, revealing also an upregulation of Cytochrome C and of death receptors (TRAILR-1, -2 and -3) and downregulation of Bcl-2 and X-linked Inhibitor of Apoptosis Protein (XIAP) (see Supplementary Fig. S1b).

### SR141716 inhibits canonical and activates non- canonical Wnt/*β*-Catenin pathway in CRC cell lines

**Figure 2:**
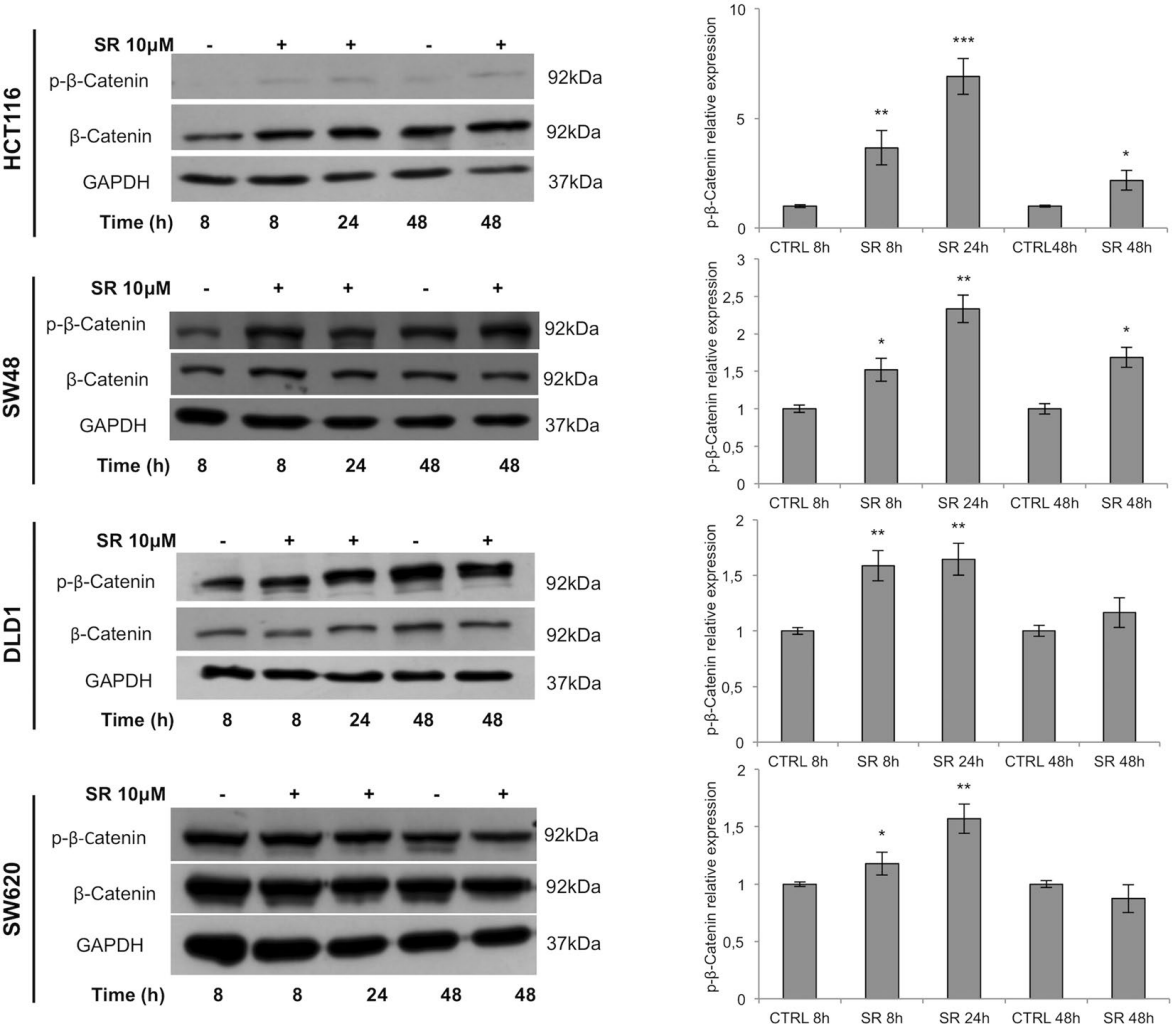
*β*-Catenin modulation in CRC cell lines. Representative western blot analysis of *β*-Catenin (total and phosphorylated form) expression in CRC cells treated with SR141716 (SR, 10 *μ*M). The histograms represent the densitometric analyses of phospho-*β*-Catenin expressed as fold change of the total *β*-Catenin amount and normalized versus GAPDH (mean ± SD; unpaired two tailed Student’s t-test *p < 0.05, **p < 0.01 and ***p < 0.005). Cropped blots from full-length gels are displayed in left panel.

**Figure 3:**
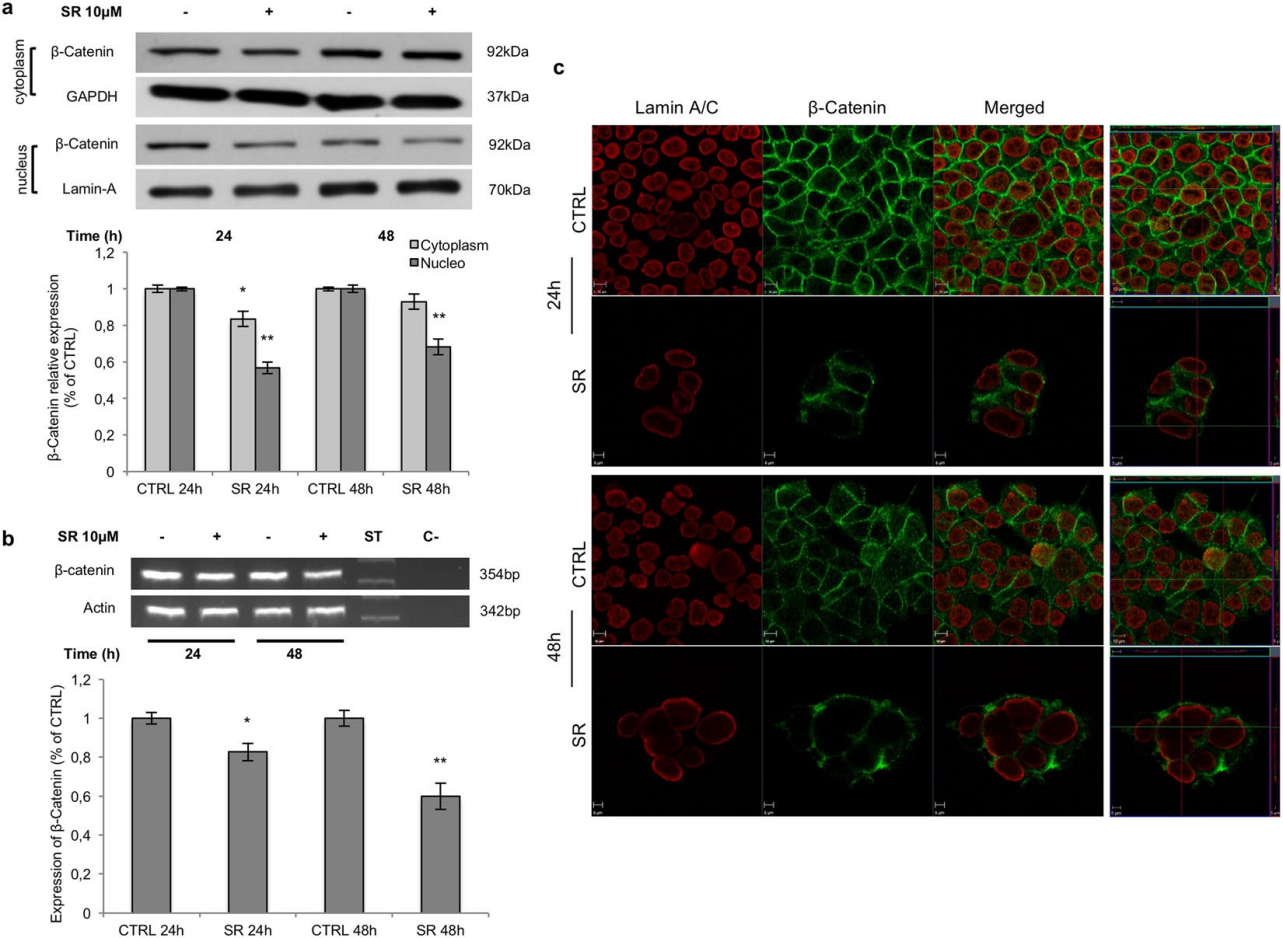
Subcellular localization of *β*-Catenin. (**a**) Western blot analysis of *β*-Catenin in nuclear and cytoplasmic fractionated extracts from HCT116 cells treated with SR141716 (SR). The histograms depict the densitometric analysis of cytoplasmic and nuclear amounts of *β*-Catenin normalized versus GAPDH and Lamin A/C respectively and expressed as percentage of control. (**b**) RT-PCR analysis of *β*-Catenin in HCT116 cells untreated (−) or treated (+) with SR141716 (SR). In the lower panel the densitometric analysis of *β*-Catenin mRNA normalized for actin is shown. Data are shown as mean ± SD (unpaired two tailed Student’s t-test *p < 0.05, **p < 0.01). (**c**) HCT116 cells treated with SR141716 (10 *μ*M) or vehicle (CTRL) were stained with anti *β*-Catenin antibody (green fluorescence) and anti Lamin A/C antibody (red fluorescence). In right panels orthogonal view of 0.5 *μ*m thickness in the z plane was reported. Cropped images from full-length gels are displayed in a and b.

**Figure 4:**
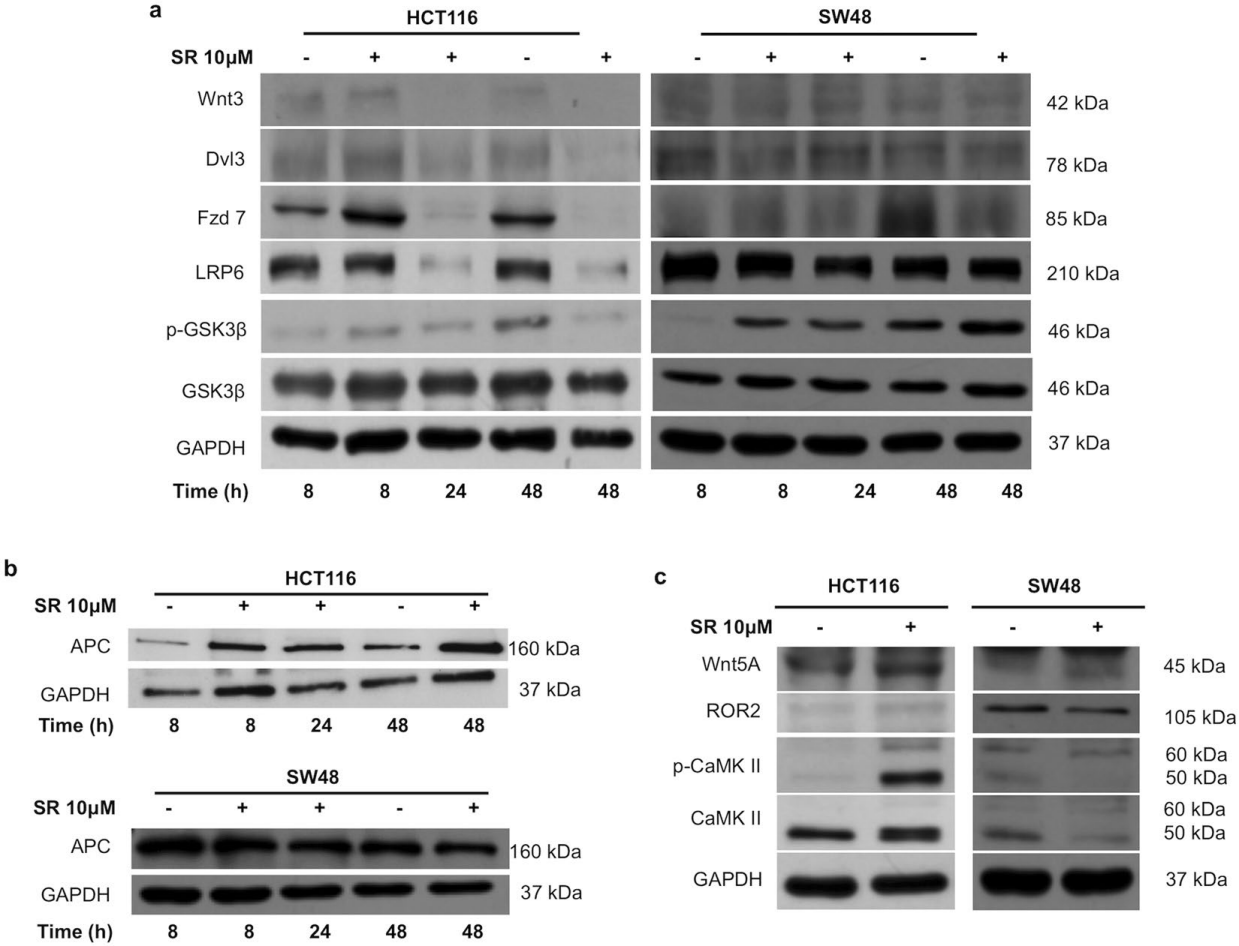
SR141716 inhibits canonical and activates non-canonical Wnt pathways in CRC cell lines. Western blot analysis for transducers of the canonical Wnt pathway (**a**) and for APC (**b**) in whole cell extracts from HCT116 and SW48 cultured for the indicated time in the presence (+) of SR141716 (SR) or vehicle alone (−). (**c**) Western blot analysis for CaMKII (total and phosphorylated), Wnt5, and ROR2 in whole cell extracts from HCT116 and SW48 cultured for 8 hours with SR141716 (+) or vehicle (−). GAPDH was used as protein loading control. Cropped blots from full-length gels are displayed in a, b and c.

In the absence of secreted Wnt ligands, the degradation complex destabilizes *β*-Catenin by phosphorylating it at Ser45 and Thr41/Ser37/Ser33. Ser33/Ser37 double phosphorylation (p-*β*-Catenin in the figures) marks *β*-Catenin for ubiquitin-mediated proteolysis^19, 20^. In HCT116 and SW48 cell lines, SR141716 increased p-*β*-Catenin starting from 8 hours of treatment (Fig. 2). To assess whether the observed effects represent a common mechanism in CRC, the expression of total and p-*β*-Catenin was also evaluated in DLD1 and SW620 cell lines where the compound induced a transient increase of the p-*β*-Catenin not persistent at 48 hours (Fig. 2). In HCT116 cell line, where the SR141716-mediated *β*-Catenin phosphorylation was more effective and persistent, the analysis of nuclear and cytoplasmic extracts from treated cells revealed a reduced amount of *β*-Catenin in the cytoplasm at 24 hours and a nuclear localization lower than control cells at both 24 and 48 hours (Fig. 3a and b). The immunofluorescence staining of SR141716-treated cells, substantially confirmed the *β*-Catenin localization and the reduction of its nuclear translocation (Fig. 3c). Wnt3 triggers the canonical *β*-Catenin signalling through the bound with Fzd7 and LRP5 or LRP6 in several cancers, including CRC^1, 21^. In HCT116, SR141716 was able to reduce Wnt3 protein levels and both Fzd7 and LRP6 co-receptor at the same time points. Furthermore, Dvl3 protein expression was inhibited by the treatment, suggesting that signal transduction across the plasma membrane and activation of Dvl3 not occurred. Despite with less extent, similar expression profiles were noticeable also in SW48 cell line, at least for Fzd7 and LRP6 (Fig. 4a and see Supplementary Fig. S2a). The *β*-Catenin phosphorylation- and degradation-complex also consists of GSK3 *β* whose activity can be inhibited by its Akt-mediated phosphorylation at Ser9^22^. We observed that SR141716 induced a precocious phosphorylation of GSK3 *β* in both cell lines, persistent until 48 hours of treatment in SW48 but not in HCT116 (Fig. 4a and see Supplementary Fig. S2b). Finally, a significant increase of APC levels was also observed in HCT116 cells (Fig. 4b and see Supplementary Fig. S2c). Wnt5 downregulation has been associated with higher tumor grade and poor prognosis^1, 5, 23^, its overexpression inhibits canonical pathway and triggers the *β*-Catenin degradation or the inhibition of TCF/Lef-mediated transcription^4, 24^. Wnt5 interacts with ROR2 (Receptor Tyrosine Kinase Like Orphan Receptor 2) tyrosine kinase receptor activating actin-binding protein, filamin A, and the JNK signalling pathway^25, 26^. However, the non-canonical pathway triggers intracellular calcium flux, associated with CaMKII activation and canonical signalling inhibition. In HCT116 cells SR141716 increased protein levels of both Wnt5A and ROR2 at 8 hours of treatment and induced activation of CaMKII (Fig. 4c and see Supplementary Fig. S3). The results strongly support an SR141716-mediated inhibition of the canonical Wnt/*β*-Catenin signalling in both HCT116 and SW48 and a drug-induced activation of the non- canonical pathway triggered by Wnt5a in HCT116 cells.

### SR141716 downregulates Wnt/*β*-Catenin target gene expression

**Figure 5:**
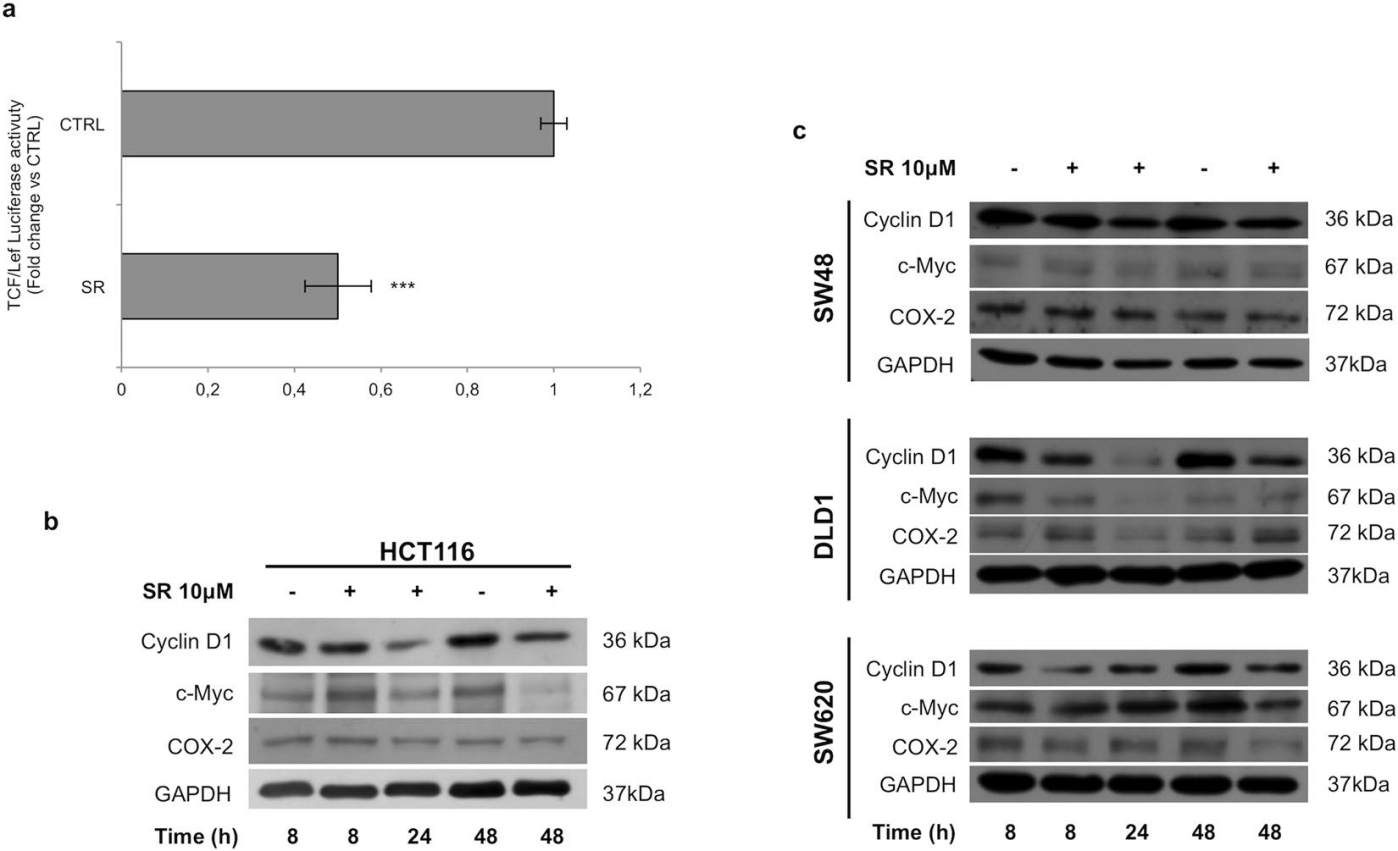
SR141716 downregulates Wnt/*β*-Catenin target gene expression. (**a**) SR141716-mediated effects on luciferase activity controlled by TRE for TCF/Lef-binding element in HCT116 cell line. Histograms represent luciferase activity measured at 18 hours from transfection in whole cell extracts from HCT116 cells transfected with reporter construct containing the TRE for TCF/Lef and treated with SR141716 (SR, 10 *μ*M) or vehicle. Firefly luciferase was normalised to Renilla luciferase reading and the data were plotted as fold change (mean ± SD; unpaired two tailed Student’s t-test ***p < 0.005) compared to control (vehicle treated) cells. Western blot analysis of Wnt/*β*-Catenin targets in HCT116 (**b**) and in CRC (**c**) cell lines untreated (−) or treated (+) with SR141716. Cropped blots from full-length gels are displayed in b and c.

Aimed to confirm that SR141716 makes *β*-Catenin unable, or at least feeble, to activate the transcription of Wnt target genes, we performed luciferase assays in HCT116 cell line. In our model, the efficiency of the transient transfection was higher than 70% (see Supplementary Fig. S4a). In HCT116 cells, transiently transfected with the reporter containing tandem repeats of a specific Transcriptional Response Element (TRE) for TCF/Lef and treated with SR141716, the luciferase activity was significantly lowered of approximately 50% compared to untreated cells (Fig. 5a). Moreover, SR141716 was able to significantly reduce protein levels of some well known Wnt/*β*-Catenin target genes, such as Cyclin D1, c-Myc (Avian myelocytomatosis virus oncogene cellular homolog) and COX-2 (Cyclooxygenase-2), involved in CRC progression (Fig. 5b and c and see Supplementary Fig. S5).

### SR141716 induces regression of CRC *in vivo*

**Figure 6:**
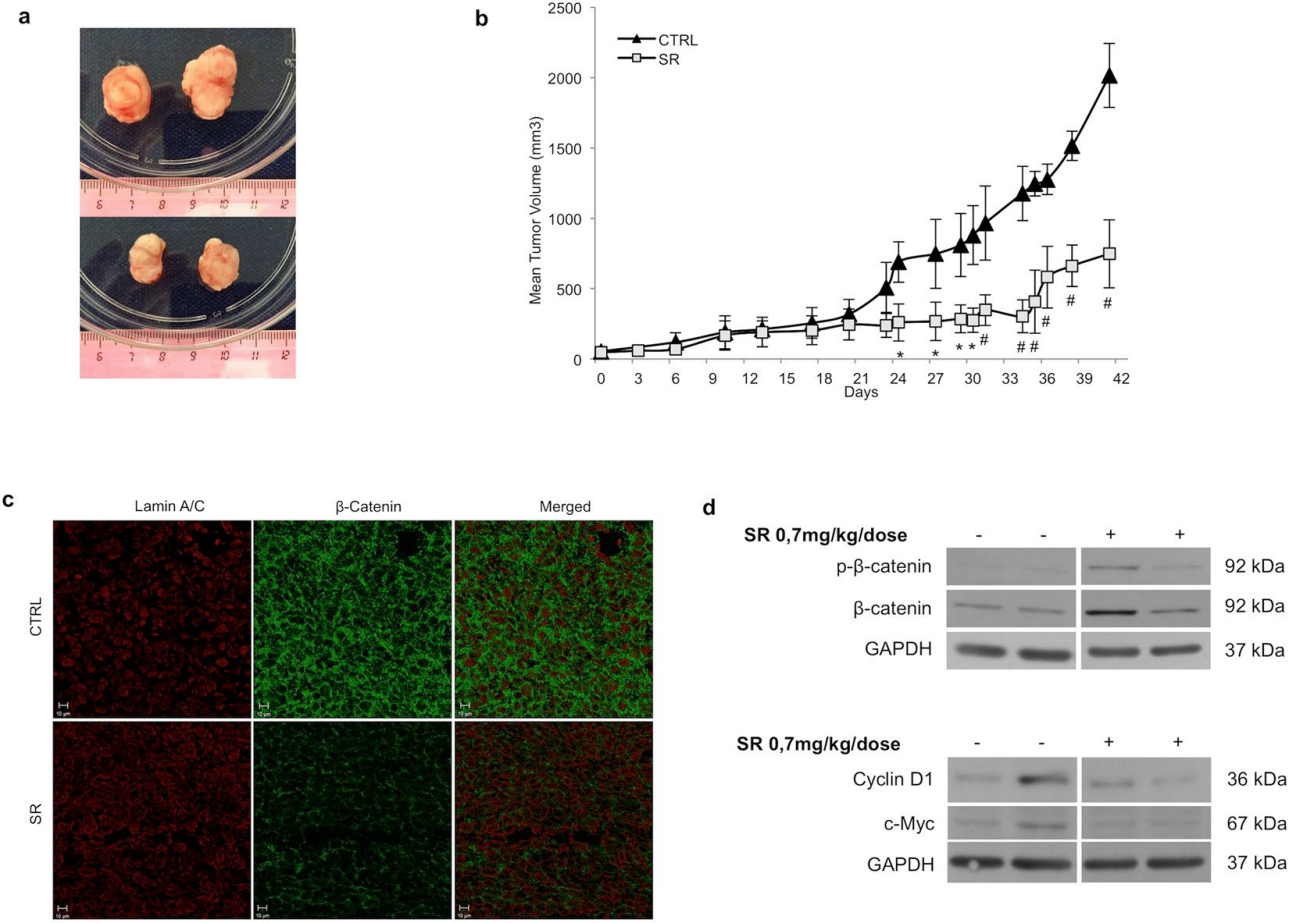
SR141716 reduces *in vivo* tumorigenicity of HCT116 through inhibition of Wnt/*β* **-**Catenin canonical pathway. (**a**) Representative growth of HCT116 xenografts in control (upper panel) and in treated groups (lower panel) at day 42. (**b**) Tumor volume growth curve after peri-tumoral injection of SR141716. Growth retardation by the compound was statistically significant for all time points labelled with * (one-way ANOVA p < 0.05) or with # (one-way ANOVA p < 0.001). (**c**) Immunofluorescence staining of HCT116 xenograft tumor sections (10 *μ*m; 42 days from treatment beginning) performed for Lamin A/C (red fluorescence) and *β*-Catenin (green fluorescence) localization. The image shown represents 3D front view; data are representative of at least three sections from each control and treated tissue sample. (**d**) Western blot analysis of total and phosphorylated *β*-Catenin, Cyclin D1 and c-Myc in whole lysate from resected tumor tissues. Cropped blots from full-length gels are displayed in d.

We tested SR141716 efficacy *in vivo* in a subcutaneous (s.c.) HCT116 xenograft model. Tumor cell suspension was injected s.c. into 20 female SCID mice and when the tumor reached approximately the size of 50–70 mm^3^, 10 mice in the treated group received the peri-tumoral injection of SR141716, while 10 mice in the control group received vehicle alone, three times a week for 6 weeks. The tumor sizes have been recorded on the first day of SR141716 treatment (day 0) and bi- or three-weekly at the indicated time points. Mice in the control group developed tumors beyond 2,0 cm^3^ on average by day 42. In contrast, the mice in SR141716 group developed much smaller tumors (Fig. 6a). In particular, starting from the thirtieth day of treatment, ANOVA analysis indicates a significant smaller tumor size in treated group compared with animals in the control group (p < 0.001) (Fig. 6b). Excised tumor sections were analyzed for *β*-Catenin localization in cellular compartments through immunofluorescence staining with specific antibodies for *β*-Catenin and for Lamin A/C (green and red fluorescence, respectively in Fig. 6c). The confocal microscopy demonstrated that in tissue sections from treated mice *β*-Catenin localized mainly in the cytoplasm whereas nuclear staining was almost devoid of specific *β*-Catenin signal (Fig. 6c and see Supplementary Fig. S6a). Finally, western blot analysis of total extracts from tissue specimens demonstrated that, despite the awaited tumor samples heterogeneity, the amount of p-*β*-Catenin, clearly detectable in the treated xenografts, was lost in control tumors. In contrast, the immunoreactivity for both Cyclin D1 and c-Myc, distinguishable in control samples, were just barely apparent in whole extracts from treated tumors (Fig. 6d). Quantification of western blot analysis, performed in samples from six tumors per condition, seems to sustain this feature even if it not reach statistical significance at least in our xenograft groups (see Supplementary Fig. S6b).

### SR141716 inhibits p300/KAT3B activity and modulates histone acetylation in CRC cell lines

**Figure 7:**
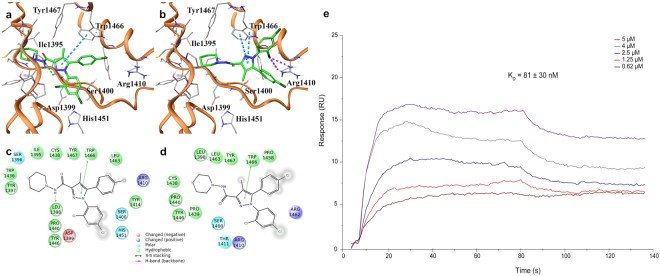
SR141716 interacts with p300/KAT3B. (**a**) 3D docking models of SR141716 (colored by atom types: C green, O red, N blue, polar H light gray, Cl dark green) in the binding site of p300/KAT3B (secondary structure depicted in orange ribbons) and (**c**) associated 2D interaction panel; (**b**) alternative 3D docking models of SR141716 and (**d**) associated 2D interaction panel. Residues in the active site are represented in sticks (colored by atom types: C grey, N blue, O red, S yellow, H light gray). H-bonds ligand/protein interactions are represented in green dotted lines, while *π*-*π* interactions are depicted with cyan dotted lines and halogen bonds are reported with violet dotted lines. (**e**) Sensorgrams obtained from different injections (0.62, 1.25, 2.5, 4 and 5 *μ*M) of SR141716 to immobilized hKAT3B/p300 (catalytic domain, aa 1284–1673).

**Figure 8:**
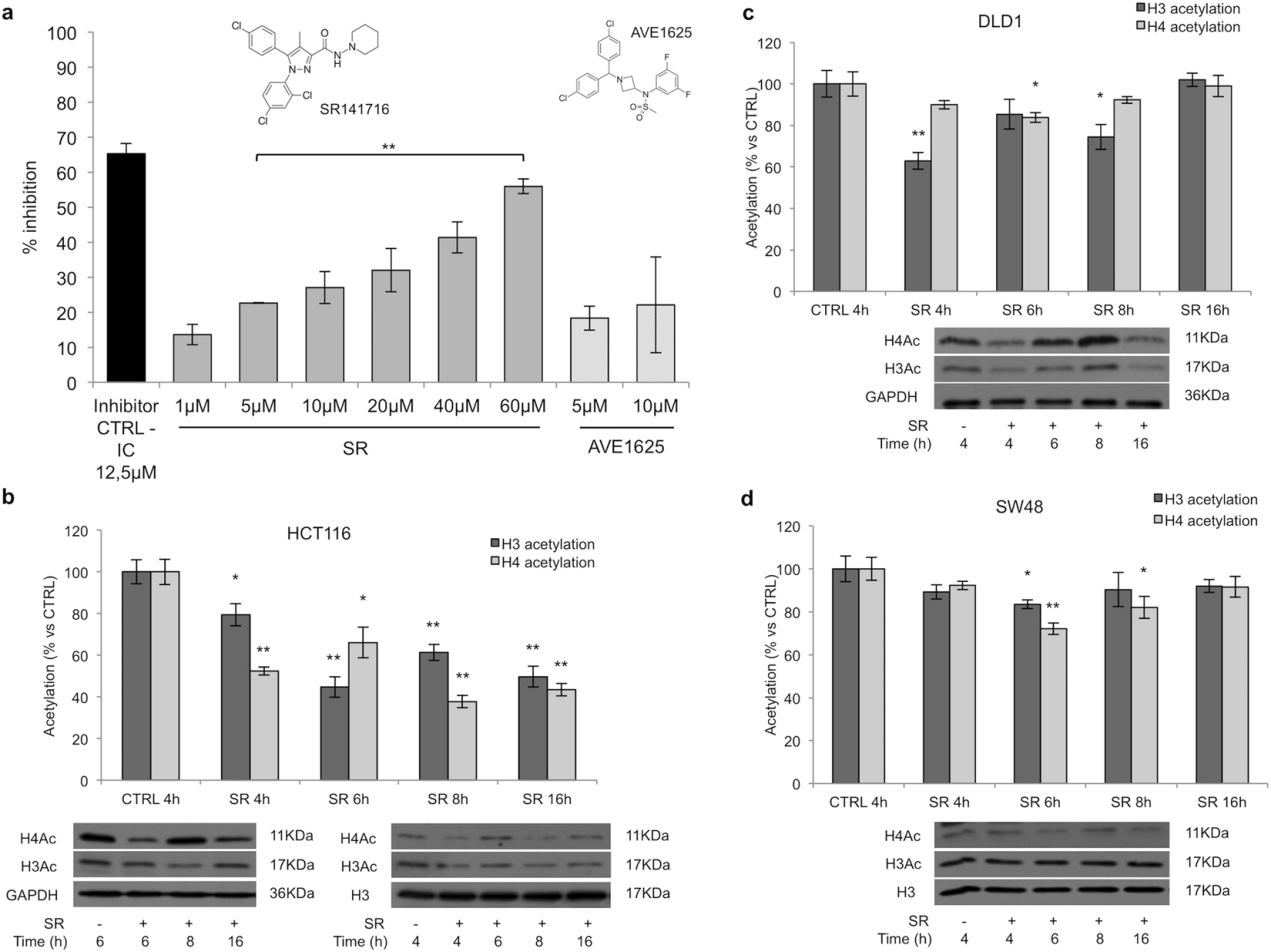
SR141716 inhibits HAT p300/KAT3B activity and decreases acetyl-histone amount in CRC cell lines. (**a**) Fluorometric assay of HAT p300/KAT3B activity performed with increasing doses of SR141716. Results were expressed as means ± SD of 3 independent experiments performed in duplicate and reported as percentage vs the enzyme control (one-way ANOVA, **p < 0.01 vs control). (**b**–**d**) Quantification of total acetyl-Histone H3 and H4 Histone extracts from CRC cell lines treated with SR141716 (10 *μ*M, SR in the figure) or with the vehicle alone (CTRL) performed with ELISA assay (histograms). Values are shown as means ± SD (n = 3) and represent the percentage of total acetylation vs control (unpaired two tailed Student’s t-test, *p < 0.05 and **p < 0.01 vs control). Lower panels: western blot analysis of purified histones (**b** on the right) and total lysates from CRC cell lines (**b** on the left, **c** and **d**) performed with anti-H4 acetylated (AcSer1, AcLys5-8-12) or anti-H3 acetylated (N-terminus) antibodies. GAPDH and histone H3 were used as loading controls. Panels are representative of 3 independent experiments. Cropped blots from full-length gels are displayed in b, c and d.

Obtained data substantially supported a drug-mediated inhibition of the canonical Wnt/*β*-Catenin signalling in CRC cell lines carrying stabilizing mutation of *β*-Catenin, such as HCT116 and SW48. Moreover, even though we found a direct inhibition of the TCF/Lef-mediated transcriptional activation in HCT116 but not in DLD1 and SW620 cell lines, in several models of human CRC the compound was able to decrease the expression of well known *β*-Catenin target genes (Fig. 5c and see Supplementary Fig. S5). We hypothesized that a member of the *β*-Catenin-TCF/Lef complex, other than *β*-Catenin, could be involved in the observed antitumor effects. Aimed to identify factors potentially able to interact with SR141716, we performed an *in silico* Inverse Virtual Screening testing the case-study compound on a panel of 306 proteins involved in cancer and inflammation events (see Supplementary Table S1). Briefly, this computational tool allows the analysis of different binding hypotheses between a single ligand and a high number of targets through molecular docking experiments, determining the selection of the most promising ligand-receptor favourite complexes after a normalization of the predicted binding affinities, and successfully directing the subsequent biological assays^27–31^. Concerning the first two identified targets (A_2A_ 1^st^ position, and ErbB4, 2^nd^ position in the ranking) (see Supplementary Table S2) we first of all considered their expression in our panel of CRC cell lines. A_2A_ (Adenosine A2a Receptor, ADORA2A) is a component of the Adenosine receptor family comprising four G-protein coupled receptors (A_1_, A_2A_, A_2B_ and A_3_) linked to Ca^2+^ mobilization and Cyclic AMP increase. Low amount of A_1_, A_2A_, and A_2B_ receptors have been detected in colon cancer cell lines cultured in normoxic condition, as in our experimental procedures^32, 33^, whereas both HCT116 and DLD1 cells expressed high levels of the A_3_ receptor subtype^32–34^. ErbB4/Her4 (Erb-B2 Receptor Tyrosine Kinase 4) is a member of the ErbB protein tyrosine kinase family, which also includes EGFR/ErbB1/Her1. Despite recently an over-expression of ErbB4 was found in human CRC tissues, in cultured colon cancer cell lines ErbB4 protein expression is difficult to detect and mainly unmistakable in poorly differentiated CRC cells such as HCT116 in our panel^35, 36^. Therefore, among the obtained results, we were intrigued by p300/KAT3B target at the 3^rd^ position in the final ranking of predicted most affine proteins of SR141716^37^. Specifically, the careful analysis of the sampled docking poses enforced this result, showing a good accommodation of SR141716 in the p300/KAT3B binding site and supporting the potential inhibition of the histone acetyltransferase (HAT) activity exerted by the investigated compound. We found two interesting binding modes in which SR141716 is placed in p300/KAT3B occupying the ligand binding site (LBD) and exerting both polar and hydrophobic interactions. The analysis of the first pose, associated to the best docking score (ΔG_bind_ = −11.2 kcal/mol), disclosed the arrangement of SR141716 in the deep part of the LBD supported by an edge-to-face *π*-*π* interaction between the pyrazole core and the indole moiety in the side chain of Trp1466, and an H-bond with the carbonyl oxygen in the backbone of Leu1398 (Fig. 7a). Further polar interactions were established with Ser1396, Asp1399, Ser1400, Arg1410, Gln1455, Lys1456, and hydrophobic contacts with Tyr1414, Leu1463, Trp1466, Tyr1467 (Fig. 7a and c). Another interesting binding mode (ΔG_bind_ = −10.8 kcal/mol) showed the placement of the molecule in a more external part of the binding site, supported by halogen bonds between the dichloro-phenyl part of SR141716 and Arg1410 (Fig. 7b), while the edge-to-face *π*-*π* interaction between the pyrazole core and Trp1466 was again detected (Fig. 7b and d). The direct binding of SR141716 to the HAT catalytic domain (aa 1284–1673) of human recombinant p300/KAT3B was corroborated by the results of a surface plasmon resonance (SPR) assay, performed according to a well-established protocol^38, 39^. In fact, Fig. 7e clearly shows a direct interaction between SR141716 and p300/KAT3B, displaying a concentration dependent SPR signal not observed with the negative control (see Supplementary Fig. S7). Fluorometric *in vitro* assay suggested a dose-dependent inhibition of p300/KAT3B HAT activity by SR141716 (5 *μ*M–60 *μ*M) (Fig. 8a). Moreover, the compound decreased the amount of acetyl-Histone H3 and acetyl-Histone H4 in both purified histones and total lysates from SR141716-treated HCT116 cells (Fig. 8b). The decreased acetylation seems a reproducible effect triggered by Rimonabant in CRC cell lines irrespective of the mutational profile and Wnt/*β*-Catenin signalling and thus can be highlighted also in DLD1 and SW48 cell lines (Fig. 8c and d).

## Discussion

The heterogeneous molecular profiles of CRC and the need to identify patients which could effectively take clinical advantage from combined chemotherapies, triggered the dissection of the mechanisms responsible for sensitivity and resistance to treatments. The majority of sporadic forms of CRC are characterized by deregulation of Wnt/*β*-Catenin signalling resulting in increased transcriptional activity of the *β*-Catenin. Despite the difficulties in to dissect the engagement of Wnt/*β*-Catenin pathway in the onset and progression of CRC, the study of these mechanisms is now emerging as a promising ground to identify potential targets of intervention for CRC treatment. The porcupine inhibitor LGK974, which blocks the palmitoylation and secretion of Wnt ligands^40^, has been recently inserted in a Phase II multi-centric clinical trial to assess the safety and antitumor efficacy of the triple combination of LGK974, LGX818 (a highly selective BRAF^V600^ inhibitor) and Cetuximab in BRAF^V600^-mutant metastatic CRC (clinical trial # NCT02278133).

Rimonabant, an inverse agonist at CB1 receptor, produced mitotic catastrophe and modulated the expression of Cyclin B1, PARP-1, Aurora B and phosphorylated p38/MAPK and Chk1, in CRC cell line^11^. The cannabinoid compounds are able to reduce the progression of CRC *in vivo* in the AOM-induced ACF model in mice^11, 14^ and improve the efficacy of chemotherapic drugs^17, 18^.

In this study, we hypothesized that cannabinoids could directly interact with the Wnt/*β*-Catenin pathway. We tested the antitumor efficacy of Rimonabant in HCT116 and SW48 CRC cell lines, expressing APC and BRAF wild type and harbouring *β*-Catenin mutation (loss of phosphorylation site S45 and S33Y, respectively) and PIK3CA activating mutations. Despite this genetic profile represents a small percentage of the human primary CRCs, the constitutive and simultaneous activation of KRAS and PIK3CA pathways, associated with *β*-Catenin stabilization, confers maximal resistance to Cetuximab^41^ and the mesenchymal phenotype reduces the sensitivity to Erlotinib^42^, features accounting for a significant number of metastatic cancers unresponsive to therapies.

In DLD1 and SW620 cells, carrying mutated form of tumor protein p53 (TP53), Rimonabant exerted cytotoxic effects without induction of apoptosis and through activation of mitotic catastrophe^11^. Unlike our previous results, here we found that Rimonabant also inhibits proliferation in TP53 wild-type highly invasive HCT116 and SW48 cell lines through induction of apoptosis clearly highlighted by an increase of Caspase 3 and PARP cleaved forms in treated cells.

Beside TP53 mutations, CRC cell lines used in this study have heterogeneous genetic features partially representatives of the broad diversity observed in CRC patients. All cells in the study, except SW48, express mutated KRAS. Allelic loss of APC has been found in SW620 and DLD1 (1338 and 1427 codon truncation, respectively) but not in APC wild-type HCT116 and SW48 cell lines which express stabilizing mutations of *β*-Catenin^43^. Finally, alterations in several tyrosine kinase genes dramatically establish the sensitivity of CRC cells and primary tumors to therapies^43^. These molecular profiles could explain the different Rimonabant-mediated effects observed in to Wnt/*β*-Catenin pathway.

In HCT116 and SW48 cells, Rimonabant induced a significant and stable increase of *β*-Catenin phosphorylation whereas this evidence seems provisional in other cell lines. These results strongly suggest the engagement of mechanisms able to antagonize *β*-Catenin phosphorylation and to induce its dephosphorylation.

The *β*-Catenin degradation complex includes protein phosphatase 2A (PP2A) and heat shock protein 105 (HSP105), two components recently identified as fine regulators of the balance between degradation and stabilization of *β*-Catenin. Although HSP105 does not possess intrinsic phosphatase activity, this protein seems essential to anchor PP2A into the degradation complex and to induce PP2A-mediated *β*-Catenin dephosphorylation^44^. In primary CRCs increased expression of HSP105 correlates with nuclear localization of *β*-Catenin and poor prognosis. Moreover, in SW480 CRC cells, characterized by stable accumulation of *β*-Catenin and truncated APC, the depletion of HSP105 decreased *β*-Catenin levels, reduced Wnt target genes expression and impaired cell proliferation through cleavage of PARP and Caspase 3, then substantially triggering apoptosis^44^. In our study we did not evaluate the HSP105 expression but we speculate that in SW620, a metastatic CRC cell line derived from the same patient originating SW480 cells, and presumably in DLD1, the transient phosphorylation of *β*-Catenin induced by Rimonabant could be ascribable to a prevailing phosphatase activity in the degradation complex.

In HCT116 and SW48, the antitumor effect of the compound was mediated by inhibition of the canonical Wnt/*β*-Catenin pathway thanks to which the stabilization of the phosphorylated form of *β*-Catenin localized the protein in the cytoplasm and inhibited its transcriptional effects. As expected, in DLD1 and, mainly, in SW620 cell lines, the Rimonabant-induced increase of p-*β*-Catenin did not seem associated to a stable inhibition of the canonical Wnt/*β*-Catenin signal, evaluated by luciferase reporter gene assay. These results originate from the different genetic profile of the used CRC cell lines and it is in agreement with the finding that APC truncations, in the SW620 and DLD1 cell lines, did not prevent *β*-Catenin phosphorylation but inhibited *β*-Catenin ubiquitination and degradation^45^. Moreover, despite all cell lines analyzed in this study expressed ROR2 receptor, LRP5 typically associated with prevalent function of the canonical Wnt/*β*-Catenin signal, was detectable in DLD1 and SW620 but not in HCT116 cells (see Supplementary Fig. S4b).

In HCT116 cell line, Rimonabant increased the activity of the non-canonical Wnt/*β*-Catenin pathway through up-regulation of Wnt5A and ROR2 receptor and activation of CaMKII. Intriguingly, the compound also raised the APC expression.

In CRCs, the loss of function of APC is mainly ascribable to truncated mutations rather than hypermethylation of the APC gene promoter^46^. On the other hand, Wnt5A is silenced in both CRC cell lines and in primary tumors due to its promoter methylation^5^. We previously demonstrated that in CRC cell lines, increased availability of cannabinoids, of both exogenous and endogenous sources, induced up-regulation of CB1-receptor expression by co-localization of PPAR *γ* and RXR *α* at the promoting region and increased the expression of *β*-ER^15^. These data allow us to hypothesize that the efficacy of the observed antitumor effects could be ascribable, at least in part, to a direct or indirect activity of Rimonabant as epigenetic modulator.

In this study, the inhibitory effects of Rimonabant were been also confirmed by decrease of the TCF/Lef transcriptional activity, in HCT116, and by a strong and persistent down-regulation of Wnt/*β*-Catenin target genes involved in the onset and progression of CRC. Surprisingly, the reduced expression of these targets seems a reproducible compound-mediated effect, but free-standing and not necessary linked to a direct inhibition of Wnt/*β*-Catenin signalling and genetic profiles of CRCs.

Despite its properties as inverse agonist at CB1 receptor, increasing evidence revealed that Rimonabant exerts CB1-independent pharmacological actions^47–49^. Aimed to identify new potential targets of the compound, we performed an *in silico* Inverse Virtual Screening on a panel of more than 300 proteins involved in cancer and inflammation and obtained results suggested at least three intriguing interactions.

Adenosine is a well known component of the tumor microenvironment and exerts pleiotropic effects in the control of tumor growth and in several phases of tumor progression such as neoangiogenesis, metastatic spreading and anti-tumor immunity^50^. The potential interaction of Rimonabant with A_2A_ receptor, suggested by results from the Inverse Virtual Screening, is a not completely surprising data, at least in the central nervous system. In striatal neurons, CB1 and A_2A_ receptors form heterodimers and functionally interact each-other in the control of pharmacological response to specific agonists^51^. Recently, in rodent model of Parkinson disease, neuroprotective effects of both Rimonabant and A_2A_ antagonist MSX3, were showed^52^. In tumors, hypoxic growth increases both adenosine and A_2A_ receptor expression^50^ and the higher tone of adenosine, released in the tumor microenvironment, induces a local immunosuppressive milieu through activation of A_2A_ receptors in substantially all effectors of the immune response, such as NK, dendritic, T_Reg_ and T cells^53^.

In the present study, CRC cell lines used for *in vitro* experiments, cultured and treated in normoxic conditions, did not express A_2A_ receptor but it is reasonable to speculate that in HCT116 xenografts Rimonabant counteracted, at least in part, the immunosuppressive stroma of the tumors and increased the anti-tumor response of immune cells, probably acting as antagonist at the A_2A_ receptor. Even though we previously demonstrated that Rimonabant is able to increase the NK cell antitumor activity in glioma^54^, a direct interaction compound-A_2A_ receptor needs to be carefully tested in immune cells and in aggressive cancer models.

ErbB4/Her4 (identified as the 2^nd^ target in the Inverse Virtual Screening ranking) and members, other than EGFR/ErbB1/Her1, belonging to the ErbB/Her family of protein-tyrosine kinases, have recently received renewed interest from the scientific community^35, 36, 55, 56^. Cetuximab, a chimeric monoclonal antibody approved for the treatment of metastatic CRCs expressing EGFR (Epidermal Growth Factor Receptor) and wild type KRAS, inhibits several transcription pathways downstream EGFR. Despite Cetuximab, used as single agent or in combination with chemotherapies, significantly improved prognosis and median survival, several patients are resistant to the compound and accumulating evidence suggests that EGFR expression, routinely tested in tumor samples with immunohistochemical assays, failed to predict the responsiveness to EGFR-targeting treatment. In a recent paper Mitsui and co-workers^36^ showed that the expression of phosphorilated ErbB family members, but not of their total forms, correlated with worse overall survival in CRC patients. Although ErbB4 protein expression is solely detectable in more aggressive CRC cell lines^35, 36^, high levels of ErbB4 were found in about 43% of primary CRC tissue microarrays^35^, and the nuclear staining of its 80kDa cleaved fragment, able to participate in transcriptional events, correlated with poor prognosis^35, 36^. In a recent work, tumor tissue specimens from patients with metastatic CRC unresponsive to cetuximab coexpressed ErbB4 and kal1 C-terminal interacting tetrasponin (KITENIN)^55^. The Authors suggested an interesting EGFR-independent interaction KITENIN/ErbB4 able to stabilize c-jun and the transcription triggered by a c-jun-TCF4-*β*-Catenin complex^55^. According to previous reports, in our study only HCT116 express both ErbB4 and KITENIN but the inhibition of Wnt/*β*-Catenin mediated transcription seems a common event in Rimonabant-treated cells probably acting downstream ErbB4 containing complex. Of course, now we are unable to exclude, at least in HCT116, a potential interaction between Rimonabant and ErbB4, but we are testing this hypothesis in *in vivo* models.

Moreover, among a large set of tested proteins, Inverse Virtual Screening *in silico* approach suggested for the first time a direct interaction of SR141716 with p300/KAT3B, that was corroborated by results from SPR-assay highlighting a direct binding of the compound to the p300-HAT catalytic domain and the significant modulation of acetyl-Histone H3 and acetyl-Histone H4 found in CRC cell lines. p300/KAT3B and CREB binding protein (CBP/KAT3A) are transcriptional coactivators that influence Wnt/*β*-Catenin signalling through specific interaction with *β*-Catenin. Despite their high homology, extensive genome-wide surveys demonstrated that the two coactivators CBP/KAT3A and p300/KAT3B exert different functions in *β*-Catenin dependent gene regulation^57, 58^. Moreover, the small molecule windorphen, a selective inhibitor of p300-HAT, showed a robust anti-tumor effect in cancer cells harbouring stable WNT signalling activation^59^.

In our study, although further experiments are needed to clarify the specific mechanisms involved and to dissect selective HAT-inhibiting properties, the observed downregulation of Cyclin-D1, COX-2 and c-Myc seems very promising data.

An impressive amount of works analyzed the role of COX-2 in the onset and progression of CRC [reviewed in ref. 60]. COX-2 is the inducible form of cyclooxygenase enzymes and guilty of the production of prostaglandins, mainly PGE_2_, involved in inflammation and tumor framework. Transcription of COX-2 gene is controlled by several consensus sequences in the promoter region, and a TCF-binding elements have been identified as functional Wnt/*β*-Catenin responsive elements within the human COX-2 promoter in both colorectal and gastric cancer cell lines^61, 62^. On the other hand, COX-2/PGE_2_ pathway can inactivate the GSK3 *β*-mediated phosphorilation of *β*-Catenin and then trigger the activation of Wnt/*β*-Catenin signalling^63^. COX-2 is frequently overexpressed in CRCs, controlled by the hypoxia-inducible factor (HIF)-1 binding to an HIF-responsive element on its promoter, and triggers the expression of proangiogenic factors such as vascular endothelial growth factor (VEGF). Moreover, COX-2-selective inhibitors exert significant antitumor effects both *in vitro* and *in vivo* ^60^.

The disregulation of the APC/*β*-Catenin axis is a frequent and early occurrence in aberrant crypt foci (ACF) induced in carcinogen treated rodent colons and in human with increased risk for developing CRC such as FAP patients. We previously found that in the mouse model of azoxymethane-induced colon carcinogenesis, Rimonabant significantly decreased the ACF formation^11^. An interesting model depicted the interplay between COX-2/PGE_2_ and *β*-Catenin during CRC induction and progression^60^. Briefly, in normoxic conditions COX-2/PGE_2_ axis promotes the stimulation of *β*-Catenin/TCF-4 activity whereas during hypoxia, a common status occurring in the advanced stage of cancer, *β*-Catenin, displaced from TCF-4, interacts with HIF-1, improves its transcriptional activity and substantially increases the expression of HIF-1 targets such as VEGF^60^. In this scenario, Rimonabant would represent a very promising compound able to counteract CRC through a direct inhibition of Wnt/*β*-Catenin pathway and COX-2/PGE_2_ axis and indirectly through the modulation of the angiogenesis according to our previous data^12^. Concerning this last process, we speculate that all together the data do not make it possible to rule out the hypothesis of a Rimonabant-mediated inhibition of coactivators directly interacting with HIF-1 such as p300/KAT3B.

Finally, our results demonstrated, for the first time, the *in vivo* efficacy of SR141716 in reducing the HCT116 xenograft growth. In tissue specimens from xenografts, the increased expression of p-*β*-Catenin and down-regulation of c-Myc and Cyclin D1 represent an encouraging validation of the Rimonabant efficacy *in vivo* in tumors harboring *β*-Catenin mutation and provide enough direct evidence for the inhibition of the canonical Wnt/*β*-Catenin pathway and of *β*-Catenin target genes by cannabinoid compounds in human CRC.

Although further experiments are needed to completely dissect the interaction of cannabinoids with Wnt/*β*-Catenin pathway, we believe that to obtain relevant clinical results in the treatment of CRCs harbouring stabilizing mutation of *β*-Catenin, Rimonabant could represent a good chance to increase the efficacy of therapies.

## Methods

### Materials

SR141716 (Rimonabant) and AVE1625 were kindly donated by Sanofi-Aventis (Montpellier, France). It was dissolved in DMSO and added to cells cultures at the indicated concentrations. Anti-*β*-Catenin, anti-Dvl3, anti-Fzd7, anti-APC, anti-Wnt5A, anti-ROR2, anti-phospho-CaMKII anti LRP5 and anti-Histone H3 were from Abcam. Anti-Cyclin D1 and anti-Lamin A/C were purchased from Becton Dickinson and Sigma-Aldrich, respectively. Anti-acetyl-Histone H4 and anti-acetyl-Histone H3 were from Santa Cruz Biotechnology and Merck Millipore, respectively. Anti-Annexin V FITC conjugated was purchased from Miltenyi Biotec. Primary antibodies not previously reported, secondary HRP-linked goat anti-mouse or goat anti-rabbit IgG, were all from Cell Signalling Technology. All the cell culture reagents were from Sigma–Aldrich, Inc.

### Cell cultures, treatments and viability assay

Human CRC cells DLD1, SW620, HCT116 and SW48 were obtained from the Interlab Cell Line Collection (IST, Genoa, Italy). Cells were routinely grown in RPMI-1640, in Dulbecco’s modified Eagle medium (DMEM), in McCoy’s 5 A or in DMEM/F12 medium, respectively, at 37 °C in a 5% CO_2_ atmosphere as described previously^15, 18^. Cells were exposed to various concentrations of SR141716 for the times showed in the figures and to evaluate cell viability the MTT assay was used as described previously^18^.

### Cell cycle and apoptosis analysis

Cells were plated in 100 mm dishes, serum starved for 18 hours to synchronize at the G_1_/S interface and treated with SR141716 (10 *μ*M) for the indicated time. Flow cytometry and FACS analysis were performed as described previously^11^. The cell cycle analysis was performed with ModFit LT v3.2 (Verity Software House, Inc.); 10000 events, corrected for debris and aggregate populations, were collected. To evaluate the apoptosis, at least 20000 events were collected, and the data were analyzed with FlowJo^®^ software (BDIS).

### Western blot analysis

Whole-cell lysates were prepared as described previously^15^. Subcellular fractionation was obtained by using NE-PER^®^ Nuclear and Cytoplasmic Extraction Reagents (Thermo scientific, Pierce biotechnology). Frozen tumor pieces from xenografts were disrupted by gentle homogenization (Potter-Elvehjem Pestle) in cold RIPA buffer. 10–30 *μ*g of proteins were loaded on SDS–polyacrylamide gels under reducing conditions and western blot analyses were performed as described previously^15^. Immunoreactive bands were quantified with Quantity One 1-D analysis software (Bio-Rad).

### Reporter gene assays

Cells were transiently cotransfected with the TCF/Lef firefly luciferase construct (100 ng) and the *Renilla* luciferase vector (10 ng) (Cignal^TM^ TCF/LEF Reporter (GFP) assay; QIAGEN). Luciferase activity was measured using a Dual-luciferase assay system (Promega) and an EnSpire-2300 luminometer (Perkin Elmer). Relative firefly luciferase activity was obtained by normalizing it to that of *Renilla* luciferase activity.

### Immunofluorescence staining

Cells were grown on slides in 12 well plates (3 × 10^4^ cells/well). After treatment, cells were fixed in parafolmadehyde (PFA, 3,7% v/v in PBS), permeabilized in Triton X-100 (0,1% v/v in PBS), blocked with 4% Bovine Serum Albumin (BSA) and incubated with anti-*β*-Catenin (Abcam) and anti-lamin A/C (Sigma-Aldrich) primary antibodies, at 4 °C overnight. Immunofluorescence staining was performed with Alexa Fluor^®^ 488 donkey anti-rabbit and Alexa Fluor^®^ 594 goat anti-mouse IgG (Molecular Probes^®^) secondary antibodies. Samples were vertically scanned from the bottom of the coverslip with a total depth of 5 *μ*m and a Plan-Apochromat oil-immersion objective (magnification 63X^*^1.7; 1.40 NA). A total of 10 z-line scans with a step distance of 0.5 *μ*m were collected and single planes or maximum-intensity z-projection of stacks and an orthogonal projection (=xy, xz, yz planes for z-stacks series) were generated.

Fixed tumor pieces from xenografts were OCT-embedded, sectioned (10 *μ*m), stained with primary and secondary antibodies (diluted in PBS/1% BSA/0,05% Triton-X-100).

A Zeiss LSM510 Laser Scanning Microscope (Carl Zeiss MicroImaging GmbH) for data acquisition was used.

### Semiquantitative RT-PCR

Total RNA extraction, cDNA synthesis and reverse-transcription PCR were performed as described^15^. Primer pairs specific to human *β*-Catenin (5′-GTCCGCATGGAAGAAATAGTTGA-3′ forward and 5′-AGCTGGTCAGCTCAACTGAAAG-3′ reverse) or to human actin (5′-ACTGGGACGACATGGAGAA-3′ forward and 5′-ATCTTCATGAGGTAGTCAGTCA-3′ reverse) were used. All reactions were performed at least in triplicate; the PCR products were quantified with Quantity One 1-D analysis software (Bio-Rad) and results were normalized to those obtained from actin B.

### Inverse virtual Screening

The chemical structures of investigated compounds (SR141716, plus 30 “blank” compounds) were built with Maestro (version 10.2) Build Panel (Maestro, version 10.2, Schr*ö*dinger, LLC, New York, NY, 2015). Prior to perform molecular docking calculations, optimizations (Conjugate Gradient, 0.05 Å convergence threshold) of the structures were applied to identify possible three-dimensional models. Then, all the structures were converted in the pdbqt format using OpenBabel software (version 2.3.2)^64^, adding Gasteiger charges.

306 protein 3D structures were prepared downloading the pdb files from the Protein Data Bank database (www.rcsb.org, see Supplementary Table S1). For each structure, “non-structural” water molecules were removed, and the processed file was then converted in.pdbqt format, merging non polar hydrogens and adding Gasteiger charges. Information about the panel of proteins are reported in Table S1.

Molecular docking calculations were performed using the Autodock-Vina software^65^. In the configuration files linked to 3D structures of the protein, coordinates and dimensions along x,y,z axes of the grid related to the site of presumable pharmacological interest, with spacing of 1.0 Å between the grid points. The exhaustiveness value was set to 64, saving 10 conformations as maximum number of binding modes. For all the investigated compounds, all open-chain bonds were treated as active torsional bonds.

A first set of promising interacting proteins of SR141716 was selected setting a predicted binding affinity cutoff = −7.5 kcal/mol. The identified proteins (166 items) were then also screened against “blank” molecules, the latter needed for the normalization^27–31^ of the binding affinities of SR141716, as reported in equation 1:

1 $$V={V}_{0}/{V}_{{\rm{R}}}$$

where, for each target investigated, V represents the normalized value of SR141716, V_0_ is its predicted binding affinity from docking calculations (kcal/mol), V_R_ is the average value of binding energy calculated on all the “blanks” (kcal/mol). It is important to note that V is a dimensionless number, and then it can be used to predict the interacting targets of a case-study compound, rather than to have precise indications about the related binding affinities. After the normalization process a final ranking was obtained, from the most to the less promising target. Normalized values and predicted binding energies for SR141716 are collected in Table S2 (see Supplementary Table S2), respectively. The ligand/protein complexes were visually inspected with Maestro (version 10.2). Illustrations of the 3D models were generated using Maestro (version 10.2).

### Surface Plasmon Resonance

SPR analyses were performed on a Biacore 3000 optical biosensor equipped with research-grade CM5 sensor chips (Biacore AB). Recombinant p300/KAT3B (Enzo Life Sciences, catalogue number *BML* − *SE*451; GenBank accession number *NM*_001429) HAT domain was immobilized (30 *μ*g/mL in 10 mM sodium acetate, pH 4.5) at a flow rate of 10 *μ*L/min by using standard amine-coupling protocols to obtain a density of 15 kRU. Myoglobin was used as negative control, and one flow cell was left empty for background subtractions. Rimonabant, dissolved in DMSO (100%), was diluted in HBS-P (50 mM HEPES pH 7.4, 150 mM NaCl, 0.005% Tween 20) and injected at 0.62, 1.25, 2.5, 4 and 5 *μ*M always maintaining a final 0.5% DMSO concentration. Binding experiments were performed at 25 °C by using a flow rate of 30 *μ*L/min, with 120 s monitoring of association and 200 s monitoring of dissociation. Regeneration of the surfaces was performed, when necessary, by a 10 s injection of 1 mM NaOH. The simple 1:1 Langmuir binding fit model of the BIAevaluation software was used for determining equilibrium dissociation constant (K_D_) from kinetic dissociation (k_d_) and association (k_a_) constants.

### Histone purification, p300/KAT3B activity assay and quantification of total Histone H3 and H4 acetylation

Total histones were extracted from cell lines (3 × 10^6^) treated with SR141716 (10 *μ*M) or vehicle alone using Histone extraction kit (Abcam) following the manufacturer’s protocol and stored at −80 °C until use. The protein concentration of the eluted histones was estimated using a Bradford protein detection kit (Bio-Rad, Hercules, CA) as previously described^15^. The p300/KAT3B enzymatic activity was performed using a KAT3B/p300 Inhibitor fluorometric assay kit (Abcam) according to the manufacturer’s protocol. Briefly, assay was performed with recombinant p300/KAT3B in the presence of SR141716, AVE1625, anacardic acid (used as inhibitor control) or vehicle alone. Histone H3 peptide and Acetyl CoA were used as acetylation substrates. The amount of fluorescence in the solution, amplified by a thiol detecting probe, was measured at Ex/Em = 392/482 nm with an EnSpire 2300 (Perkin Elmer) multimode plate reader. The results were expressed as percentage of inhibition compared to the enzyme control. Quantification of total acetyl-Histone H3 and H4 was performed in histone extracts, purified as earlier described, through a colorimetric ELISA assay, and in total nuclear extracts, using western blot assay. In both analysis, extracts from cell lines treated with SR141716 (10 *μ*M) or with the vehicle alone, for the times shown in the figures, were evaluated in at last three independent experiments.

### *In vivo* studies

#### Animals

20 female SCID mice (SHO, 6–8 weeks old) were obtained from Charles River, maintained under clean room conditions in sterile filter top cages with Alpha-Dri bedding and housed on high-efficiency particulate air (HEPA)-filtered ventilated racks. Animals received sterile rodent chow and water *ad libitum*. All experimental procedures were conducted in accordance with the Institute for Laboratory Animal Research Guide for the Care and Use of Laboratory Animals. The experimental protocols received the approval of the Ethical Committee of the Italian Board of Health (prot. n. 0031993 and n. 0031994, June, 6, 2013).

#### Subcutaneous xenograft models in athymic mice

HCT116 cells (1 × 10^6^, suspended in 150 *μ*l of PBS) were implanted subcutaneously into the right flank region of each mouse and allowed to grow to the size of approximately 50–70 mm^3^. SR141716 (0,7 mg/kg/dose in 150 *μ*l of PBS/20% Glycerol) was administered three time a week by peri-tumoral injection for 6 weeks. Animals in the corresponding control group received PBS/20% Glycerol (150 *μ*l) injected on the same schedule as SR141716. Mice were daily monitored for clinical signs and mortality. The dose of SR141716 was selected on the basis of previous studies where the compound showed to exert effects *in vivo* on tumor xenografts without reduction of spontaneous activity, impaired locomotion, signs of wasting or toxicity^66^. Progress of tumors was determined by two dimensional caliper measurements, and tumor volumes were calculated using a standard hemi ellipsoid formula: [length (mm) × width (mm)^2^]/2. Tumor volumes were analyzed using one-way ANOVA. At the end of the study, mice were humanely euthanized and tumors were resected and frozen immediately or fixed in 10% formalin for further analysis.

### Statistical analysis

Data obtained from multiple experiments were calculated as means ± SD, if not otherwise specified, and analyzed for statistical significance by using the two tailed Student t-test, 1- or 2-way ANOVA for independent groups, with the Tukey or Bonferroni correction for multiple comparisons. All data shown are representative of at least three independent experiments performed in triplicate. Values of p < 0.05 were considered statistically significant.

### Data availability statement

All data generated or analysed during this study are included in this published article (and its Supplementary Information files).

## Electronic supplementary material

Supplementary Information
