# Supplementary material for: Inhibition of Wnt/β-Catenin pathway and Histone acetyltransferase activity by Rimonabant: a therapeutic target for colon cancer
Source: Sci Rep. 2017 Sep 15;7:11678. doi: 10.1038/s41598-017-11688-x (PMC5601949; doi:10.1038/s41598-017-11688-x)
Supplement: Supplementary file 1 — Supplementary Information [file 41598_2017_11688_MOESM1_ESM.pdf]

Supplementary information

Inhibition of Wnt/β-Catenin pathway and Histone acetyltransferase activity by Rimonabant: a therapeutic target for colon cancer.

M. C. Proto[1], D. Fiore[1], C. Piscopo[1], S. Franceschelli[1], V. Bizzarro[1], C. Laezza[3], G. Lauro[1], A. Feoli[1], A. Tosco[1], G. Bifulco[1], G. Sbardella[1], M. Bifulco[2] and P. Gazzerro[1§].

[1]Department of Pharmacy, University of Salerno, Via Giovanni Paolo II, 84084 Fisciano, Salerno, Italy

[2]Department of Medicine and Surgery, University of Salerno, Via Salvatore Allende, 84081 Baronissi Salerno, Italy

[3]Institute of Endocrinology and Experimental Oncology, IEOS CNR, Via Pansini 5, 80131 Naples, Italy;

[§] Corresponding Author:

Patrizia Gazzerro, PhD
Assistant Professor in General Pathology
Department of Pharmacy -University of Salerno
Via Giovanni Paolo II, 132
84084 Fisciano (SA) - Italy
phone/fax: +39-089-969452/+39-089-969602
mail to: pgazzerro@unisa.it

Table S1 The panel of 306 targets used in this study, with specified information about the grid used for docking calculations.[a]

Target	PDB code	Center coordinates			Size of grid along axis		
		x	y	z	x	y	z
14_3_3_epsilon	2BR9	-17.799	2.054	20.299	16	16	22
14_3_3_gamma	2B05	-68.008	-35.843	-65.126	22	16	14
14_3_3_sigma	1YWT	26.322	42.314	62.000	22	16	14
14_3_3_theta	2BTP	28.243	-20.467	33.117	22	18	16
14_3_3_zeta	1QJA	20.373	-11.190	43.447	20	16	18
17hsd3	3KLM	-3.194	5.222	-12.194	18	16	22
CPA	1CBX	-1.042	33.136	-6.050	21	21	21
CPU	3D67	41.827	38.616	42.591	26	24	20
EPHa3	3DZQ	-6.623	16.238	56.390	20	20	22
EPHb4	2X9F	12.385	6.033	11.642	20	20	20
a2a_ago	2YDV	32.098	7.921	-17.681	16	24	24
a2a_ant	3EML	-9.394	-9.004	52.769	18	24	28
abl2	3HMI	-30.312	8.541	-15.143	18	14	18
abl	2HYY	16.615	60.851	42.083	36	38	36
aif	1M6I	2.760	51.263	23.172	20	14	28
akap13	2LG1	6.045	1.351	-0.883	54	42	44
akt1	3MVH	17.474	-1.885	27.396	20	20	20
akt2	3D0E	21.279	-18.979	8.086	20	22	24
alk5	2WOU	2.423	8.157	4.369	20	16	16
alk	2XP2	29.411	46.982	8.863	16	16	16
alk_alternative	3AOX	-18.736	13.648	-3.639	26	28	28
aman2	3DDF	29.685	64.280	10.770	24	24	32
ape1	2ISI	30.330	10.876	32.821	16	16	16
arf6	2W83	43.175	0.447	15.647	20	22	22
arid3a	2KK0	2.951	-3.134	-1.349	58	52	44
ask1	3VW6	-2.118	7.449	27.148	22	22	22
atad2	3DAI	-12.463	41.138	3.561	56	52	52
aurkinA	2W1D	2.999	34.523	3.684	22	22	22
aurkinB	2VG0	10.028	-0.449	2.834	24	22	22
bap1	2W15	10.534	12.859	21.170	14	14	14
bcl2a1	2VM6	-4.000	9.651	58.187	26	16	16
bcl2	2O21	3.249	5.600	3.161	16	16	16
bcl6	3LBZ	-4.609	2.416	20.623	22	20	20
bclw	1ZY3	1.000	-10.592	2.873	26	22	16
bclxl	1BXL	4.000	-11.550	1.062	24	16	16
brca	4IGK	13.608	-14.986	10.561	26	26	26
brd2_bd1	2YDW	23.395	11.443	-11.573	18	18	16
brd2_bd2	3ONI	44.077	42.044	-9.953	16	16	16
brd3_bd1	3S91	46.055	5.436	12.948	20	18	18

brd3_bd2	3S92	32.502	24.434	17.841	20	18	18
brd4_bd1	3MXF	30.178	15.747	-1.351	20	20	20
brd4_bd2	2YEM	-5.795	-3.216	-17.316	20	20	20
brd9_4nqn	4NQN	-15.756	14.283	-14.566	18	22	22
brd9_alt	5IGN	61.555	-2.412	24.086	18	20	18
brd9	4XY9	59.389	-4.155	25.545	16	18	14
brdt	4KCX	28.538	-24.103	5.784	16	20	24
btk	3PIX	19.034	7.855	-3.847	22	16	20
bubr1k	3SI5	13.993	7.667	21.534	30	24	24
cSRC	3F3V	-4.939	33.779	-7.177	24	20	22
cabp7	2WOR	0.197	5.214	5.850	22	16	30
calmodulin	3EWT	26.150	16.896	41.536	20	20	20
camKIIB	3BHH	19.194	68.631	32.637	22	24	22
caspase1	2FQQ	52.000	28.170	39.946	16	14	12
caspase2	1PYO	-2.478	24.046	27.105	22	18	18
caspase3	3EDQ	-7.472	-8.883	9.361	16	16	18
caspase7	1SHL	52.800	13.900	5.432	30	30	30
caspase8	1QTN	-9.591	32.000	43.500	16	16	18
cathepsinB	1GMY	31.019	35.506	35.000	12	22	24
cathepsinG	1AU8	13.299	41.721	3.414	16	18	24
cathepsinK	2R6N	32.090	7.855	3.945	20	16	16
cathepsinL	3HWN	33.000	-27.000	-12.966	24	18	16
ca_2	2X7S	21.428	32.153	19.042	24	24	24
cbfa2t1	2OD1	-1.262	0.080	-1.406	36	42	48
cbp	2RNY	-21.832	-3.546	-4.244	38	26	26
cbp_brd	4NR7	19.487	27.582	-2.837	20	20	20
cbp_brd_wat	4NR7	19.487	27.582	-2.837	20	20	20
cdc42_no_alf3	2NGR	27.969	0.399	9.383	24	18	20
cdk2	2VV9	1.153	25.016	8.894	16	18	16
cdk2_altern	2WFY	41.893	25.613	1.032	18	18	18
cdk2_alt	2WIH	28.753	5.411	61.065	20	20	20
cdk4	3G33	-44.623	-23.874	-58.372	32	30	28
cdk5	1H4L	31.964	-19.457	34.006	30	32	30
cdk6	2F2C	30.249	22.000	60.500	14	16	12
cdk7	1UA2	42.707	-3.136	23.335	30	30	30
cdk8	3RGF	-6.073	2.926	28.602	38	38	40
cdk9	3BLQ	49.918	-19.584	-11.700	16	14	14
chetk_alpha	4GC8	22.040	-10.419	8.546	20	26	22
chk1	2QHN	17.400	-2.427	10.285	16	14	14
chk2	2W7X	46.710	-14.819	-10.824	16	16	22
ciap1	3D9U	6.049	16.721	11.491	14	20	16
ck2	3FL5	23.097	6.627	17.825	20	22	22
ck2_alt	3PE1	21.455	-32.592	18.621	20	28	26
clk1	1Z57	5.753	12.504	22.896	30	30	30
clk3	2WU6	36.404	59.008	16.400	12	16	16
cmet	2WGJ	21.882	80.521	3.407	14	14	18
cox_1	3N8X	-18.561	-49.009	-1.877	24	22	22

cox_1_3kk6	3KK6	-31.141	42.768	-7.323	22	22	22
cox_2	3NT1	-42.655	-52.344	-23.765	22	24	20
cxcr4	2K05	-5.086	-3.516	3.094	20	24	22
cyp19a1	4GL5	84.199	53.199	51.321	22	26	28
dal1	3BIN	47.246	74.157	0.758	24	18	30
dapk	3EH9	-21.618	1.723	-12.865	22	20	18
dcr3	3K51	-18.656	7.259	35.418	30	22	40
ddb1	3I7P	19.163	37.871	41.208	26	22	24
ddx53	3IUY	-1.479	8.845	31.644	18	14	18
dhfr	1PD8	30.179	15.695	-0.515	22	20	22
diaminoox	3HIG	-30.099	-11.287	74.580	14	14	20
dlc2	2H80	2.363	-5.667	3.381	52	42	42
dnmt3a	3A1B	34.666	-6.035	13.225	24	24	20
dnmt3l	2QRV	60.000	100.605	19.871	20	30	12
dyrk_1a	3ANQ	4.690	28.024	12.200	26	22	26
egfr	2J6M	-50.000	0.373	-18.696	14	12	22
ejc	3EX7	-32.961	11.428	27.119	22	24	20
enolase1_gr1	2PSN	81.332	-20.050	-6.650	22	20	18
enolase1_gr2	2PSN	60.597	-19.041	20.178	20	24	18
enolase2_gr1	1TE6	46.142	36.062	24.468	22	22	24
enolase2_gr2	1TE6	31.119	51.339	3.344	22	20	26
enolase3_gr1	2XSX	-32.828	12.580	2.201	22	26	22
enolase3_gr2	2XSX	0.684	4.118	-3.291	22	22	18
erbB2	1S78	18.505	27.000	214.145	20	30	20
erbB4	2R4B	-14.146	11.917	-3.129	20	20	20
erbB4_alt	3BBT	-37.517	49.803	16.476	28	18	34
erk1	2ZOQ	28.863	6.828	17.411	30	30	30
erk2	2OJG	-13.188	13.830	41.302	22	16	18
errA	3K6P	36.477	-1.714	35.201	20	24	20
errB_ant	homology_modeling	19.541	42.088	29.426	20	20	22
errB_apo	homology_modeling	-16.371	-5.373	-29.356	20	20	20
errB_gsk	homology_modeling	-53.704	121.991	2.500	22	22	22
errB_gsk_h2o	homology_modeling	-53.704	121.991	2.500	22	22	22
errG_ant	1S9Q	19.659	42.116	30.043	18	18	20
errG_apo	2E2R	-16.371	-5.373	-29.356	20	22	20
errG_gsk	2GPP	-53.703	122.879	1.944	20	24	22
errG_gsk_h2o	2GPP	-53.703	122.879	1.944	20	24	22
e-cadherin	2O72	40.500	12.000	55.000	30	30	30
fadk1	3BZ3	10.246	1.881	2.017	24	20	26
fak	3BZ3	9.988	3.459	4.865	16	16	16
fgf1	1HKN	-19.795	-5.075	36.612	16	16	16
fgfr1	1AGW	8.727	4.577	19.000	14	10	20
fgfr2	2PVF	21.792	-6.259	-2.777	18	16	18
flt3	3QS7	-3.435	37.259	-61.682	40	26	28
fpps_allo	3N1V	4.461	78.308	28.791	22	20	20
fpps_ipp	1ZW5	13.086	33.007	-6.741	18	18	18
fpps_no_ipp	1ZW5	11.806	33.007	-6.741	22	18	20

fscn1	3LLP	30.918	10.585	7.519	22	22	22
ftase	1LD8	17.110	135.160	-1.069	16	14	16
fxr	1OSV	21.179	42.946	12.608	26	24	20
galectin1	1W6M	0.871	60.097	25.245	16	16	12
galectin3	1KJL	-1.495	-7.002	0.865	20	16	12
galectin7	3GAL	5.147	8.086	28.917	16	12	16
grb7	2QMS	32.870	19.585	46.920	18	26	16
gsk3	3F7Z	0.590	13.013	17.987	30	30	30
gstm2_2	3GUR	10.202	-7.431	16.808	26	24	26
gstp1	2A2R	10.846	7.407	26.035	14	14	14
haspin	3E7V	12.731	-10.431	-10.993	24	20	22
hdac1	4BKX	-44.755	16.619	-5.512	16	16	24
hdac2	3MAX	69.400	33.500	-0.297	18	20	20
hdac3	4A69	34.423	60.188	26.892	23	29	25
hdac4	2VQM	16.376	-10.087	2.960	18	24	34
hdac6	homology_modeling	18.908	-9.000	-0.126	20	22	23
hdac7	3C0Z	-35.000	-32.700	-23.000	27	27	27
hdac8b	1VKG	21.859	69.000	72.500	23	23	23
hdac8	1T64	107.700	59.300	26.383	24	24	27
hgfr_cmet_alt	2WD1	104.332	21.278	140.166	20	20	18
hla1	3BO8	-19.175	19.257	-4.369	22	42	22
hmt	3HNA	-10.912	-1.472	24.020	32	20	22
hras	2UZI	6.723	25.453	26.054	18	20	22
hsc70	3FZH	17.063	-4.127	-0.134	20	20	22
hsp70_allo_site	3JXU	5.808	-30.125	18.937	14	14	12
hsp90	2WI6	34.663	9.291	27.661	16	14	14
hspa1a	3JXU	-5.677	-17.671	16.741	26	28	26
hspa1lA	3GDQ	12.887	9.377	7.092	22	34	32
hspa1lB	3GDQ	12.887	9.377	7.092	22	34	32
hspa2	3I33	-15.025	-5.569	7.634	28	26	24
hspa5	3IUC	-6.578	-0.465	1.186	34	32	30
hspa6A	3FE1	-2.351	-4.413	21.062	28	26	24
hspa6B	3FE1	-2.351	-4.413	21.062	28	26	24
ido	2D0T	60.151	53.354	19.000	36	36	36
igf	3F5P	80.817	51.567	149.088	26	20	16
irak4	2NRU	30.739	6.930	-2.308	20	20	24
jak1	3EYG	12.000	13.903	-14.249	20	16	16
jak2	3E64	33.332	34.394	37.000	14	22	14
jak3	1YVJ	8.374	-13.359	-7.996	16	16	16
jmjd3	2XXZ	45.578	45.880	4.878	15	15	15
jmjd3_4ask	4ASK	44.186	44.880	7.000	20	20	20
jmjd3_akg	2XXZ	40.000	45.910	5.455	18	18	18
jnk1	4IZY	-13.979	5.567	-22.334	24	28	22
jnk2	3NPC	53.345	47.600	9.200	32	34	36
jnk3	2ZDT	47.427	6.999	31.001	22	24	18
jnk3_alt	3KVX	-25.515	32.554	12.483	28	18	22
kga	3VOY	-5.021	18.201	16.327	24	24	28

kif11	3CJO	18.757	15.434	107.995	22	18	24
kif15	4BN2	-21.874	16.722	-51.467	20	24	20
kit_kinase	3G0E	33.268	-4.448	-76.442	30	30	30
kit_kinase_alternative	4HVS	21.659	-2.264	7.637	24	28	20
lck	3AD4	-25.151	-1.321	-7.567	24	20	20
lox	3O8Y	3.055	17.715	-0.739	22	26	22
lsd1	2EJR	23.606	44.189	50.526	26	32	30
lyn	2ZVA	18.550	-10.780	25.417	24	22	28
mcl1	3D7V	12.500	-22.982	22.500	26	26	26
mdm2	3EQS	-3.945	-2.024	6.550	22	28	18
mdmx	3EQY	-15.391	19.122	15.766	28	20	16
mdmx_alternative	4N5T	9.882	21.234	-14.170	20	26	20
mek1	3DV3	39.338	-11.778	-1.356	16	18	18
mek1_adp	1S9J	32.376	29.688	39.434	24	24	18
mek1_no_adp_no_lig	1S9J	32.376	29.688	39.434	24	24	18
mek2_adp	1S9I	38.839	101.877	46.799	32	24	30
mek2_no_adp_no_lig	1S9I	38.839	101.877	46.799	26	18	24
mek4_anp	3ALN	16.902	-1.048	-26.289	22	20	20
mek4_no_anp	3ALN	16.902	-1.048	-26.289	22	20	20
mek5_pb1_domain	1WI0	-0.416	-0.860	-0.263	46	46	46
metap2	1YW9	16.517	30.297	20.282	16	16	20
mhp37	3E78	30.969	32.012	31.058	30	22	22
mif	3B9S	-28.479	12.851	-15.782	24	22	20
mk2	3M42	36.903	2.935	15.901	18	18	20
mlk1	3DTC	21.917	38.957	32.260	22	22	22
ml_iap	3F7H	79.515	65.841	16.928	30	20	20
mmp3	1HY7	-1.500	49.563	56.892	18	12	26
mmp8	2OY2	-11.500	-3.134	5.371	26	28	30
mmp13	830C	-5.196	26.703	52.731	14	14	20
mpges_1_4al1	4AL1	8.554	-9.254	-4.804	20	20	24
mpges_1_no_gsh	4AL0	10.304	-11.033	-8.384	24	20	30
mpges_1_with_gsh	4AL0	10.304	-11.033	-8.384	24	20	30
mps1	4H7X	49.683	54.589	56.182	32	30	36
mrp1	2CBZ	-18.000	47.285	3.763	18	14	18
msk1	3KN5	24.505	37.471	76.124	18	24	20
mthfs	3HY3	4.247	15.887	-10.368	18	20	18
mtor	3FAP	-9.212	26.369	36.057	20	20	20
mtsp1	3NCL	21.607	28.392	27.485	20	20	20
nek2	2XKF	-27.131	19.628	-18.874	20	20	22
nek7	2WQN	-9.772	-31.792	-46.457	20	18	20
nm23_h2	3BBB	-24.262	-21.020	45.862	18	26	24
nnos	3JT4	11.695	0.011	25.550	16	16	16
nq02	3TZB	17.082	1.632	-6.666	24	24	28
nqo1	2F1O	10.513	11.320	-4.000	16	16	20
nr3c4	2Q7K	25.465	2.178	4.975	26	26	24
orf73	2YPY	0.743	45.269	21.521	24	18	32
p38	3HEG	0.206	2.797	20.549	24	28	30

p53_mut	2X0V	125.393	103.818	-42.832	18	18	16
p300	3BIY	-17.116	17.800	2.628	18	32	16
palb2	3EU7	-11.743	-8.240	6.425	26	24	26
pan_bcr_abl	3IK3	3.533	-1.334	16.867	28	28	22
parp	2JVN	-1.605	-0.821	4.533	40	48	50
pcaf	2RNW	13.278	-1.754	2.797	16	24	18
pcna	1VYJ	-43.984	66.000	36.537	20	20	20
pd1_chain_b	3BIK	8.343	-3.970	-25.030	40	22	30
pd1_chain_c	3BIK	4.343	-30.970	-4.030	36	30	28
pdk1	3NAX	5.483	-3.504	9.156	34	34	34
pdk2	4MP2	10.608	3.673	-37.761	20	20	22
pd	3BIK	2.876	-14.538	-25.455	22	24	24
peroxiredoxin_1	2RII	-6.836	9.736	-13.002	14	14	18
peroxiredoxin_hORF6	1PRX	18.838	2.806	1.665	20	22	22
pgm	1YFK	3.188	-2.533	53.526	14	14	14
pi3k	3ENE	45.329	12.738	29.269	22	22	24
pig3	2J8Z	26.715	19.374	-18.999	22	22	22
pimkin	3JYA	5.477	82.059	1.200	36	36	36
pka	3L9L	29.205	3.090	8.632	18	20	18
pka_c_alpha	4AXA	6.566	10.987	0.767	22	26	22
pkb_alpha	2UVM	16.822	7.994	18.563	18	22	18
pkca	3IW4	5.236	29.781	51.904	16	16	16
pkcbII	2I0E	38.888	55.638	34.802	16	16	20
pkct	2JED	58.284	15.628	-3.697	16	16	20
pkc_eta	3TXO	21.080	11.783	16.450	24	20	20
pkc_iota	3A8W	5.500	-21.705	-12.402	22	24	24
pkc_iota_2	1ZRZ	-8.918	20.387	20.039	24	24	22
pkc_iota_apo	3A8X	20.567	3.010	-43.634	28	24	20
pkm2	4G1N	0.453	-12.672	47.009	30	30	28
plk1	3FVH	0.655	12.362	9.376	22	12	14
pnk	2W3O	28.835	-5.080	-23.878	16	22	22
pop	3DDU	-4.757	13.470	34.000	20	16	20
ppar_g	3AN3	18.921	73.753	11.153	24	26	24
prp31	2OZB	-5.633	-29.445	10.077	30	28	28
pten	1D5R	32.000	87.025	24.255	14	14	12
pxr	1M13	14.282	74.983	0.974	30	36	32
pyk2	3FZS	-2.502	-2.819	13.265	20	20	20
rac1	4GZL	-10.630	-6.059	12.325	28	20	18
rack1	4AOW	-7.151	-63.398	-5.935	22	20	40
raf	3C4C	6.651	24.264	34.675	36	36	36
rar	4DQM	-2.149	-21.501	14.594	20	14	26
ras	3GFT	11.333	33.635	19.939	22	18	20
ret	2X2K	-9.887	8.110	10.108	22	18	18
rihb	3B9X	-16.483	13.405	15.473	24	24	24
ring3	4FLP	11.621	6.748	-53.930	20	22	20
rnase_a	4G8V	34.391	0.156	11.313	22	34	28
rock1	3TWJ	16.506	33.311	15.984	18	20	26

rxr	4M8H	61.454	46.549	30.542	24	14	22
s6k_alpha1	2Z7Q	2.918	-2.632	23.470	22	26	22
sirt1	4I5I	46.030	-26.747	18.672	26	32	26
soyb_lox	1HU9	25.999	3.901	12.148	26	22	32
srpk	1WBP	20.000	39.793	7.000	16	20	14
stat1_s1	1YVL	-15.427	-1.000	126.502	22	26	22
stat1_s2	1YVL	-17.904	-15.031	114.432	26	24	24
stat3	1BG1	96.616	73.201	68.027	22	22	22
strad_alpha	3GNI	2.435	-20.980	-28.236	26	20	28
survivin	1XOX	-25.152	-39.750	-77.198	24	24	36
syk	3FQH	-4.383	-5.583	10.308	20	20	18
tank1	2RF5	-13.446	-20.332	-24.937	20	20	22
tank2	3KR8	38.766	-11.747	-12.114	16	16	18
tao2	2GCD	45.644	56.288	0.502	22	20	20
tdp1	1RFF	7.194	54.070	-1.204	36	36	36
tie2	2OO8	54.000	2.432	73.772	14	16	20
tip1	2L4T	-7.260	-6.905	6.757	32	26	32
top2B_3qx3	3QX3	33.969	94.096	50.851	28	30	26
top2B_4g0v	4G0V	33.969	94.096	50.851	28	30	26
topII	1ZXM	28.160	33.381	32.264	30	30	30
topII_atp	1QZR	35.098	-0.762	38.389	22	20	22
topI	1K4T	21.005	-3.741	28.466	16	22	10
tp	1UOU	-4.500	2.335	27.725	28	24	28
ts	3NCL	0.231	13.226	17.549	16	20	16
upa	2VIP	27.065	8.579	28.568	18	16	16
vegfr1	1FLT	19.812	-3.886	8.346	24	28	30
vegfr2	3EWH	14.078	-6.092	8.682	36	36	36
wee1	1X8B	4.307	53.947	25.912	16	16	16
wif1	2YGN	6.798	-14.342	16.307	28	26	16
xiap	1TFT	-6.421	-11.133	3.653	20	18	18
zap70	1U59	11.166	5.172	54.018	22	18	20
zf21	2RRF	1.475	-2.295	-0.414	56	48	42

a. After visual inspection, we noticed that the reported grid dimensions were too small for some “blanks”, causing poor predicted binding affinity values. In these cases (cutoff = -4.0 kcal/mol), we enlarged the grid dimensions of the specific target of 6 points along the x,y,z axes.

Table S2. Final ranking of 166 interacting targets of SR141716, from the highest to the lowest normalized V values, and related original predicted binding affinities before normalization. The averaged predicted binding affinity calculated for the 30 “blanks” against each target is reported in the last column. The remaining 140 targets from the original panel of 306 items were not considered for the normalization due to the poor predicted binding affinities with SR141716 (cutoff=-7.5 kcal/mol).

Position in the ranking	Target	PDB code	V	Predicted binding affinity of SR141716 (kcal/mol)	Averaged predicted binding affinity calculated for "blanks" (kcal/mol)
1	a2a_ant	3EML	1.256	-11.4	-9.1
2	erbB4	2R4B	1.221	-10.2	-8.4
3	p300	3BIY	1.214	-11.2	-9.2
4	upa	2VIP	1.197	-9.1	-7.6
5	brd9	4XY9	1.196	-8.8	-7.4
6	topI	1K4T	1.183	-11.0	-9.3
7	chk2	2W7X	1.182	-10.4	-8.8
8	mlk1	3DTC	1.179	-10.8	-9.2
9	mek1_adp	1S9J	1.166	-9.6	-8.2
10	jak1	3EYG	1.163	-9.4	-8.1
11	alk5	2WOU	1.143	-9.7	-8.5
12	lsd1	2EJR	1.128	-9.9	-8.8
13	lsd1_FAD	2EJR	1.127	-9.9	-8.8
14	cdk2_alt	2WIH	1.126	-9.9	-8.8
15	dcr3	3K51	1.118	-7.6	-6.8
16	enolase3_gr1	2XSX	1.102	-7.5	-6.8
17	lyn	2ZVA	1.100	-9.3	-8.5
18	pkc_eta	3TXO	1.100	-10.0	-9.1
19	camKIIB	3BHH	1.097	-9.1	-8.3
20	mmp13	830C	1.093	-9.7	-8.9
21	cdk2	2VV9	1.092	-9.5	-8.7
22	abl	2HYY	1.092	-9.5	-8.7
23	ring3	4FLP	1.091	-8.9	-8.2
24	ppar_g	3AN3	1.087	-9.4	-8.6
25	CPU	3D67	1.083	-8.9	-8.2
26	pkct	2JED	1.083	-10.4	-9.6
27	hsp90	2WI6	1.080	-8.7	-8.1
28	aif	1M6I	1.080	-10.7	-9.9
29	mek1_no_adp_no_lig	1S9J	1.074	-9.2	-8.6
30	EPHb4	2X9F	1.071	-9.1	-8.5
31	jnk3	2ZDT	1.071	-9.0	-8.4
32	s6k_alpha1	2Z7Q	1.071	-8.8	-8.2
33	alk	2XP2	1.068	-8.3	-7.8

34	erbB4_alt	3BBT	1.066	-9.3	-8.7
35	survivin	1XOX	1.065	-9.0	-8.5
36	brd9_alt	5IGN	1.065	-8.5	-8.0
37	atad2	3DAI	1.065	-8.0	-7.5
38	pkca	3IW4	1.064	-9.4	-8.8
39	akt1	3MVH	1.063	-9.7	-9.1
40	clk1	1Z57	1.062	-9.7	-9.1
41	msk1	3KN5	1.062	-9.2	-8.7
42	igf	3F5P	1.062	-8.8	-8.3
43	bap1	2W15	1.059	-8.4	-7.9
44	gsk3	3F7Z	1.057	-9.0	-8.5
45	mmp8	2OY2	1.055	-9.3	-8.8
46	tank2	3KR8	1.054	-8.3	-7.9
47	metap2	1YW9	1.053	-8.7	-8.3
48	calmodulin	3EWT	1.053	-8.2	-7.8
49	mdm2	3EQS	1.052	-8.0	-7.6
50	pcaf	2RNW	1.050	-8.1	-7.7
51	fgfr2	2PVF	1.049	-9.2	-8.8
52	mk2	3M42	1.048	-8.1	-7.7
53	zap70	1U59	1.047	-9.0	-8.6
54	pyk2	3FZS	1.046	-8.7	-8.3
55	fak	3BZ3	1.046	-8.6	-8.2
56	clk3	2WU6	1.045	-9.7	-9.3
57	mek1	3DV3	1.045	-8.9	-8.5
58	top2B	3QX3	1.043	-9.8	-9.4
59	hsc70	3FZH	1.043	-8.9	-8.5
60	17hsd3	3KLM	1.040	-7.7	-7.4
61	ftase	1LD8	1.039	-8.9	-8.6
62	hla1	3BO8	1.038	-8.3	-8.0
63	jnk1	4IZY	1.036	-8.3	-8.0
64	hdac2	3MAX	1.034	-7.9	-7.6
65	tdp1	1RFF	1.033	-7.7	-7.5
66	hdac8b	1VKG	1.031	-9.1	-8.8
67	cdk9	3BLQ	1.029	-8.4	-8.2
68	mps1	4H7X	1.028	-7.7	-7.5
69	dapk	3EH9	1.027	-8.9	-8.7
70	pkc_iota_apo	3A8X	1.026	-9.1	-8.9
71	wif1	2YGN	1.025	-9.8	-9.6
72	pkc_iota_2	1ZRZ	1.025	-8.8	-8.6
73	ca_2	2X7S	1.024	-8.7	-8.5
74	hspa6B	3FE1	1.023	-7.6	-7.4
75	EPHa3	3DZQ	1.022	-8.2	-8.0
76	pop	3DDU	1.022	-7.9	-7.7
77	nq02	3TZB	1.021	-9.6	-9.4
78	dyrk_1a	3ANQ	1.021	-9.4	-9.2
79	pimkin	3JYA	1.021	-8.9	-8.7
80	pkc_iota	3A8W	1.021	-8.7	-8.5

81	mtor	3FAP	1.021	-10.4	-10.2
82	CPA	1CBX	1.019	-7.7	-7.6
83	lck	3AD4	1.018	-8.2	-8.1
84	ck2_alt	3PE1	1.018	-8.1	-8.0
85	topII	1ZXM	1.018	-7.9	-7.8
86	haspin	3E7V	1.017	-8.7	-8.6
87	fadk1	3BZ3	1.017	-8.6	-8.5
88	irak4	2NRU	1.016	-9.3	-9.2
89	cmet	2WGJ	1.015	-8.9	-8.8
90	mthfs	3HY3	1.015	-8.0	-7.9
91	brd3_bd1	3S91	1.013	-7.8	-7.7
92	caspase3	3EDQ	1.011	-7.6	-7.5
93	pka_c_alpha	4AXA	1.010	-9.6	-9.5
94	rack1	4AOW	1.010	-9.1	-9.0
95	cdk7	1UA2	1.009	-9.1	-9.0
96	bclw	1ZY3	1.008	-9.1	-9.0
97	srpk	1WBP	1.008	-7.9	-7.8
98	fpps_allo	3N1V	1.007	-8.1	-8.0
99	caspase7	1SHL	1.003	-8.8	-8.8
100	jnk3_alt	3KVX	1.002	-8.3	-8.3
101	brdt	4KCX	1.002	-8.3	-8.3
102	ret	2X2K	1.001	-8.1	-8.1
103	hspa1lB	3GDQ	0.998	-8.3	-8.3
104	fgfr1	1AGW	0.998	-7.9	-7.9
105	btk	3PIX	0.995	-8.0	-8.0
106	nek2	2XKF	0.994	-8.0	-8.0
107	mek2_no_adp_no_lig	1S9I	0.992	-8.3	-8.4
108	rar	4DQM	0.990	-8.6	-8.7
109	mtsp1	3NCL	0.990	-8.4	-8.5
110	pdk1	3NAX	0.989	-8.7	-8.8
111	jmjd3_akg	2XXZ	0.989	-7.7	-7.8
112	pkcbII	2I0E	0.988	-8.7	-8.8
113	brd9_4nqn	4NQN	0.988	-8.0	-8.1
114	mpges_1_4al1	4AL1	0.987	-8.9	-9.0
115	ts	3NCL	0.987	-8.6	-8.7
116	topII_atp	1QZR	0.985	-9.1	-9.2
117	rock1	3TWJ	0.985	-8.3	-8.4
118	jak3	1YVJ	0.981	-8.3	-8.5
119	p38	3HEG	0.981	-8.1	-8.3
120	top2B_4g0v	4G0V	0.975	-8.7	-8.9
121	mek4_no_anp	3ALN	0.975	-8.3	-8.5
122	cox_2	3NT1	0.974	-8.0	-8.2
123	gstp1	2A2R	0.972	-7.6	-7.8
124	pka	3L9L	0.965	-9.4	-9.7
125	a2a_ago	2YDV	0.965	-9.0	-9.3
126	erk2	2OJG	0.965	-8.1	-8.4
127	brd4_bd2	2YEM	0.965	-7.9	-8.2

128	mmp3	1HY7	0.964	-8.7	-9.0
129	cox_1_3kk6	3KK6	0.963	-7.5	-7.8
130	jak2	3E64	0.962	-8.6	-8.9
131	cSRC	3F3V	0.962	-8.5	-8.8
132	kga	3VOY	0.956	-8.0	-8.4
133	cdk4	3G33	0.956	-7.6	-8.0
134	chetk_alpha	4GC8	0.951	-8.0	-8.4
135	aurkinA	2W1D	0.951	-7.9	-8.3
136	erk1	2ZOQ	0.950	-8.2	-8.6
137	cdk5	1H4L	0.948	-7.9	-8.3
138	pi3k	3ENE	0.944	-8.1	-8.6
139	mhp37	3E78	0.943	-8.3	-8.8
140	brd3_bd2	3S92	0.943	-7.5	-8.0
141	kif11	3CJO	0.941	-8.2	-8.7
142	pan_bcr_abl	3IK3	0.941	-8.1	-8.6
143	bcl2	2O21	0.939	-7.7	-8.2
144	errG_ant	1S9Q	0.934	-8.5	-9.1
145	diaminoox	3HIG	0.932	-8.3	-8.9
146	bclxl	1BXL	0.929	-7.6	-8.2
147	tao2	2GCD	0.928	-8.2	-8.8
148	arid3a	2KK0	0.928	-7.5	-8.1
149	jmjd3	2XXZ	0.927	-7.5	-8.1
150	ejc	3EX7	0.926	-8.4	-9.1
151	mpges_1_no_gsh	4AL0	0.920	-8.0	-8.7
152	vegfr2	3EWH	0.915	-8.2	-9.0
153	strad_alpha	3GNI	0.910	-7.8	-8.6
154	hspa2	3I33	0.909	-8.4	-9.2
155	wee1	1X8B	0.909	-8.1	-8.9
156	pxr	1M13	0.906	-8.7	-9.6
157	fpps_no_ipp	1ZW5	0.902	-7.5	-8.3
158	hmt	3HNA	0.898	-7.9	-8.8
159	cdk8	3RGF	0.898	-7.5	-8.4
160	nnos	3JT4	0.892	-8.4	-9.4
161	dhfr	1PD8	0.890	-8.6	-9.7
162	ras	3GFT	0.890	-7.9	-8.9
163	sirt1	4I5I	0.885	-8.3	-9.4
164	jnk2	3NPC	0.885	-7.7	-8.7
165	pkm2	4G1N	0.812	-7.9	-9.7
166	errB_ant	homology_modeling	0.797	-7.7	-9.7

Supplementary information

Figure legends

Supplementary Figure S1. Effect of SR141716 treatment on the apoptotic protein expression profiles in CRC cell lines.

(**a**), Quantitative analysis of representative western blots, showed in Figure 1d, carried out for cleaved Caspase 3 and cleaved PARP in HCT116 and SW48 cells treated with SR141716 (SR, 10µM). Results (arbitrary units; cleaved form/total form/GAPDH) were expressed as mean ± SD of three independent experiments (unpaired two tailed Student's *t*-test *p<0,05, **p<0,01, ***p<0,005 *vs* control). (**b**), Cells were treated with SR141716 (SR, 10µM) for 48h and cell lysates were used to evaluate the protein expression through a Human Apoptosis Antibody Array (Abcam- Cambridge, UK). Left panel shows representative proteomic membrane pictures. Experiments were performed in duplicate. The squares highlighted the significantly modulated proteins not previously analyzed by western blot assay. In the right panel, densitometric analysis of the membrane spots was reported. The analysis was obtained through TotalLab™ software and array data normalization was performed according to manufacturer's instructions. Data were expressed as mean ± SD (unpaired two tailed Student's *t*-test *p<0,05 *vs* control)

Supplementary Figure S2. Effect of SR141716 on canonical Wnt/β-Catenin pathway.

Quantitative analysis of representative western blots in Figure 4a showing Wnt3, Dvl3, Fzd7 LRP6 (**a**) and p-GSK3β (**b**) protein levels in HCT116 and SW48 treated with SR141716 or vehicle alone. (**c**), Quantitative analysis of representative western blot for APC showed in Figure 4b and carried out on whole cell extracts from indicated cell lines treated with the compound (SR, 10µM). Results (arbitrary units) were expressed as mean ± SD of three independent experiments (unpaired two tailed Student's *t*-test *p<0,05, **p<0,01, ***p<0,005 *vs* control).

Supplementary Figure S3. Effect of SR141716 on non canonical Wnt/β-Catenin pathway.

Quantitative analysis of representative western blots reported in Figure 4c showing Wnt5A and ROR2 protein expression in HCT116 (**a**) and SW48 (**b**) treated with SR141716 (SR, 10μM) or vehicle. (**c**), Quantitative analysis of representative western blot for pCaMKII showed in Figure 4c Results (arbitrary units; phosphorylated form/total form/GAPDH) were expressed as mean ± SD of at least three independent experiments (unpaired two tailed Student's *t*-test *p<0,05, **p<0,01, ***p<0,005 *vs* control).

Supplementary Figure S4. Transfection efficiency of luciferase assay and expression of ROR2 and LRP5 receptors in CRC cell lines.

(**a**), Left panel: Fluorescence microphotographs of HCT116 cells transfected with a constitutively expressing GFP reporter construct, used as positive control in the luciferase assays (magnification 10x). Right panel: efficiency of the transfection procedure was calculated as percentage of green cells *versus* total cells (mean of cellular counts performed in at least 5 microphotographs from 4 independent experiments ± SD); unpaired two tailed Student's *t*-test **p<0,01. (**b**) Representative western blots and densitometric analysis of ROR2 and LRP5 receptors constitutively expressed in CRC cell lines used in the study. Results (arbitrary units; receptor/GAPDH) were expressed as mean ± SD. Cropped blots from full-length gels are displayed in b.

Supplementary Figure S5. SR141716-induced downregulation of Wnt/β-Catenin target genes.

Quantitative analysis of representative western blot depicted in Figure 5b and 5c for Cyclin D1, c-Myc, COX-2 in total extracts from HCT116 (**a**), SW48 (**b**), DLD1 (**c**) and SW620 (**d**) treated with the compound (SR, 10μM) for the indicated time. Results (arbitrary units) were expressed as mean ± SD (unpaired two tailed Student's *t*-test *p<0,05, **p<0,01, ***p<0,005 *vs* control).

Supplementary Figure S6. β-Catenin localization and target genes expression in tumor sample from HCT116 xenograft.

(**a**), Fixed tumor pieces were OCT-embedded, sectioned and labeled with fluorescent antibody against β-Catenin (green fluorescence). Nuclei were stained with Lamin A/C (red fluorescence). Localization of β-Catenin was analyzed in the orthogonal view of 0.5 µm thickness in the z plane. Data are representative of at least three experiments with similar results. (**b**), Quantification of western blot analysis for p-β-Catenin (left panel), Cyclin D1 and c-Myc (right panel) in tumors (n=6 per condition) from HCT116 xenografts s.c. treated with SR141716 or PBS/Glycerol for 42 weeks. Results (arbitrary units) were expressed as mean ± SEM.

Supplementary Figure S7. SPR interaction analysis of SR141716 binding to immobilized control protein.

Sensograms obtained from different injections (0.62, 1.25, 2.5, 4 and 5 µM) of SR141716 to immobilized myoglobin.

Supplementary Figure S8. Uncropped images for western blots displayed in main figure 1d.

Supplementary Figure S9. Uncropped images for western blots displayed in main figure 2.

Supplementary Figure S10. Uncropped images for western blots and gels displayed in main figure 3a and 3b, respectively.

Supplementary Figure S11. Uncropped images for western blots displayed in main figure 4a (left panel).

Supplementary Figure S12. Uncropped images for western blots displayed in main figure 4a (right panel).

Supplementary Figure S13. Uncropped images for western blots displayed in main figure 4b.

Supplementary Figure S14. Uncropped images for western blots displayed in main figure 4c.

Supplementary Figure S15. Uncropped images for western blots displayed in main figure 5b and 5c (upper panel).

Supplementary Figure S16. Uncropped images for western blots displayed in main figure 5c (central and lower panel).

Supplementary Figure S17. Uncropped images for western blots displayed in main figure 6d.

Supplementary Figure S18. Uncropped images for western blots displayed in main figure 8.

Supplementary Figure S19. Uncropped images for western blots displayed in supplementary figure 4b.

a

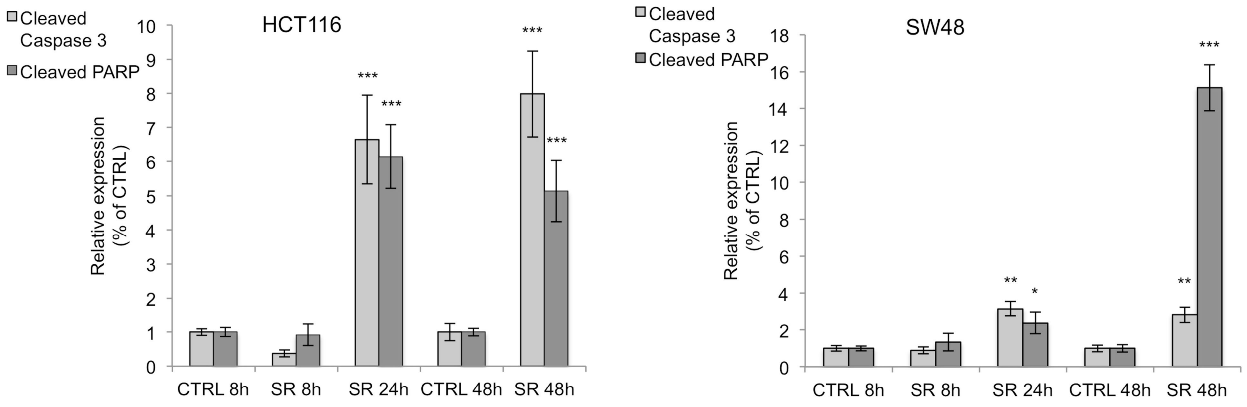

b

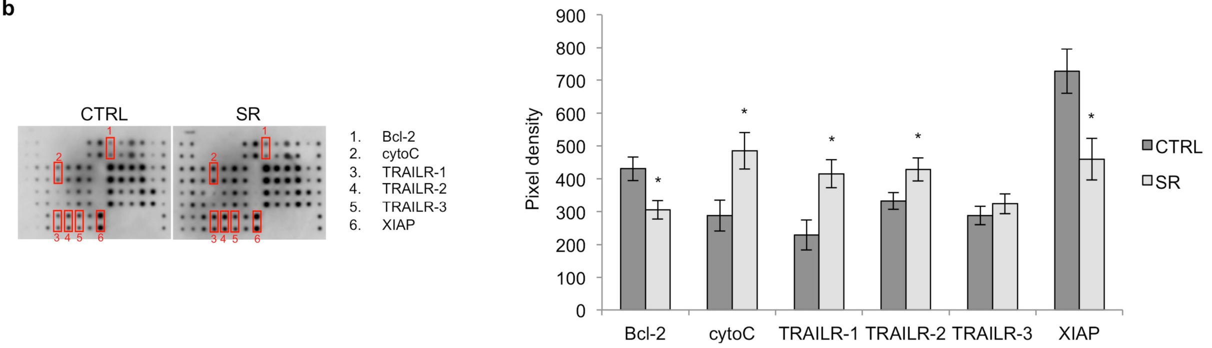

Supplementary Figure S1

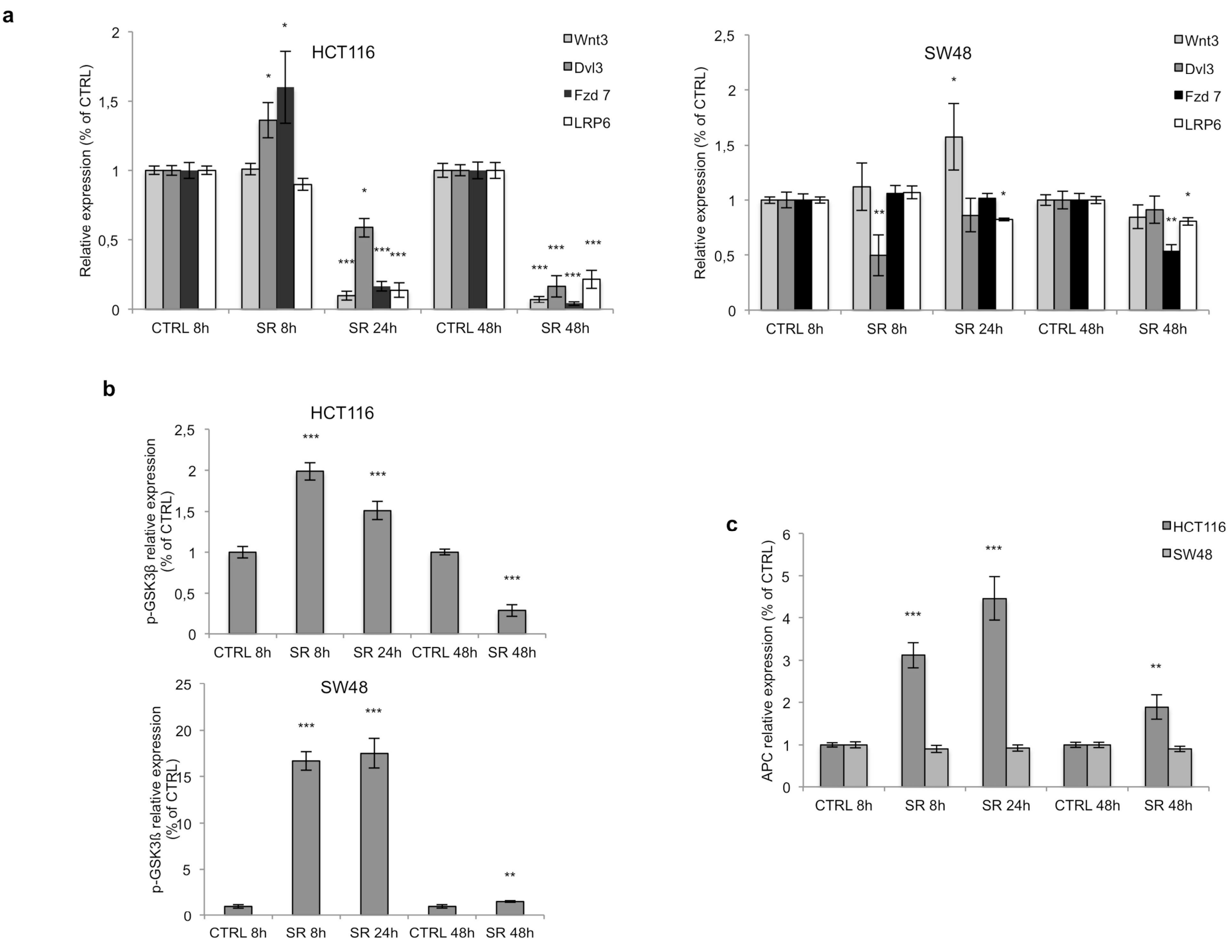

Supplementary Figure S2

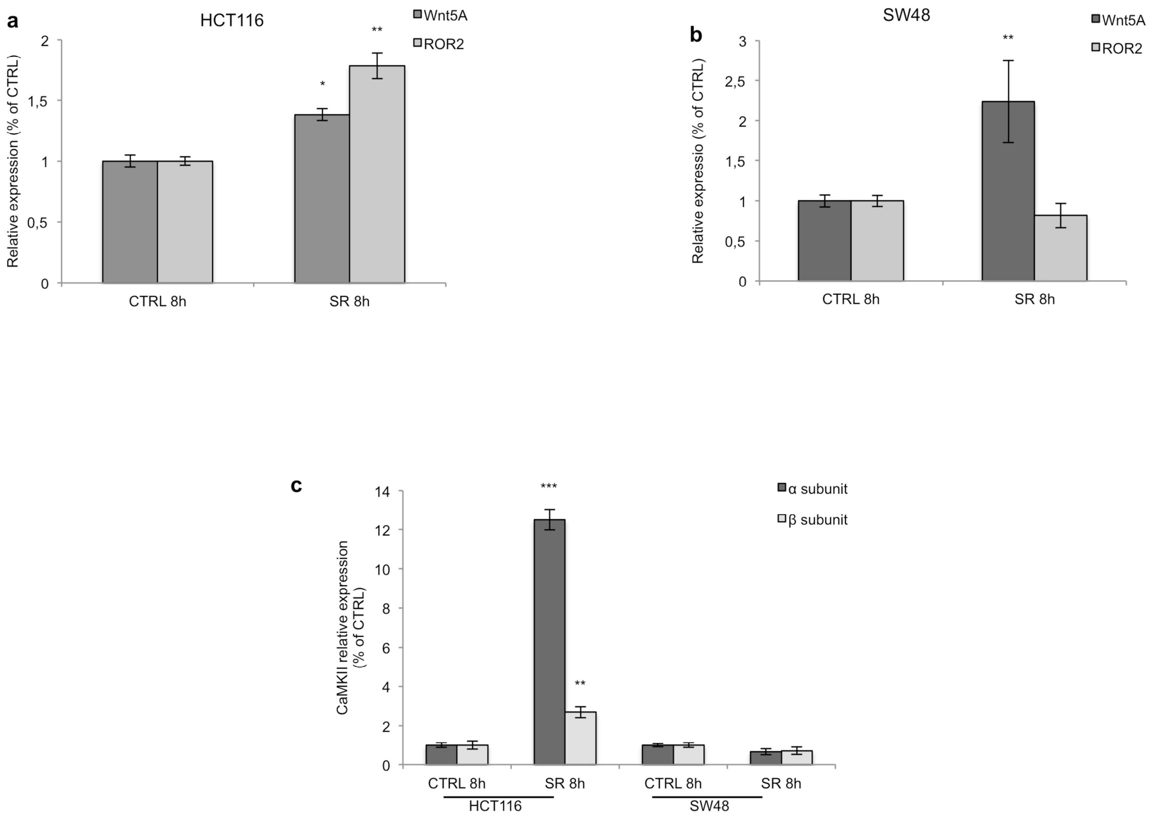

Supplementary Figure S3

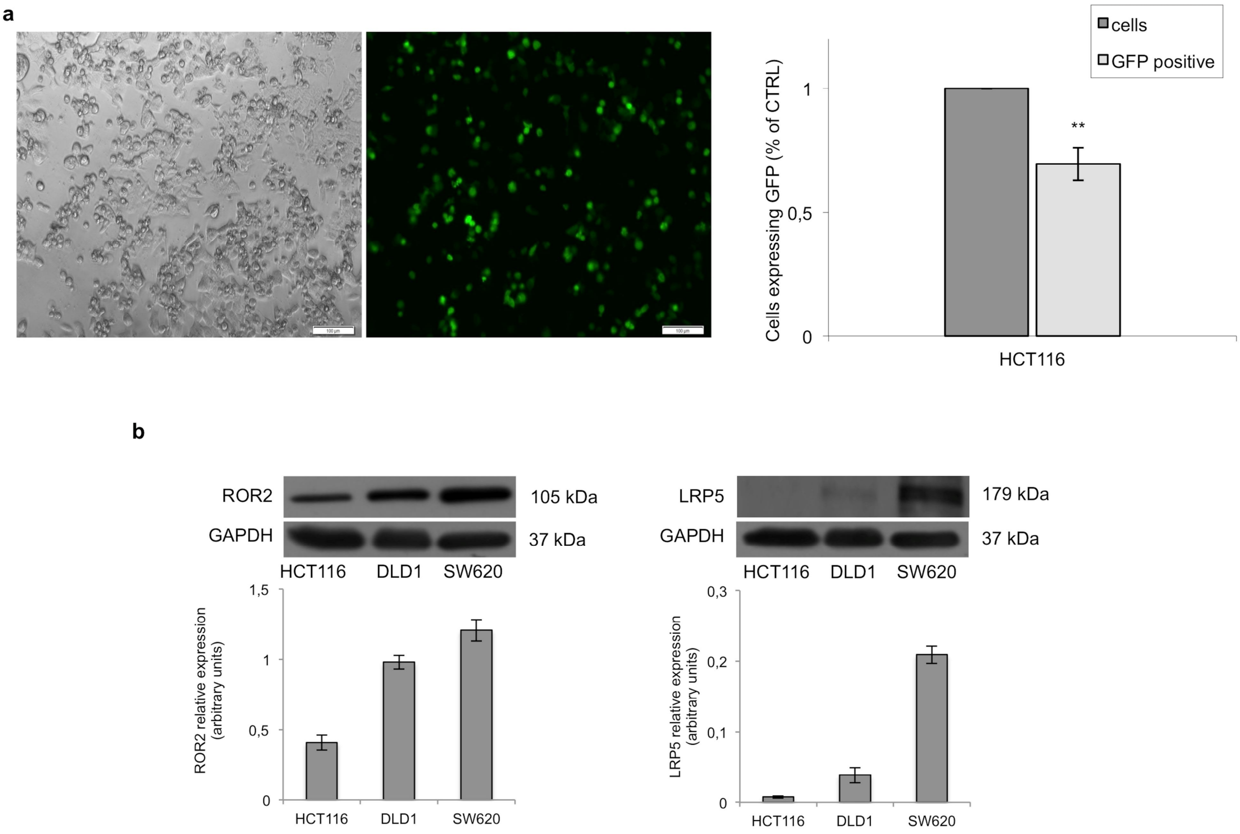

Supplementary Figure S4

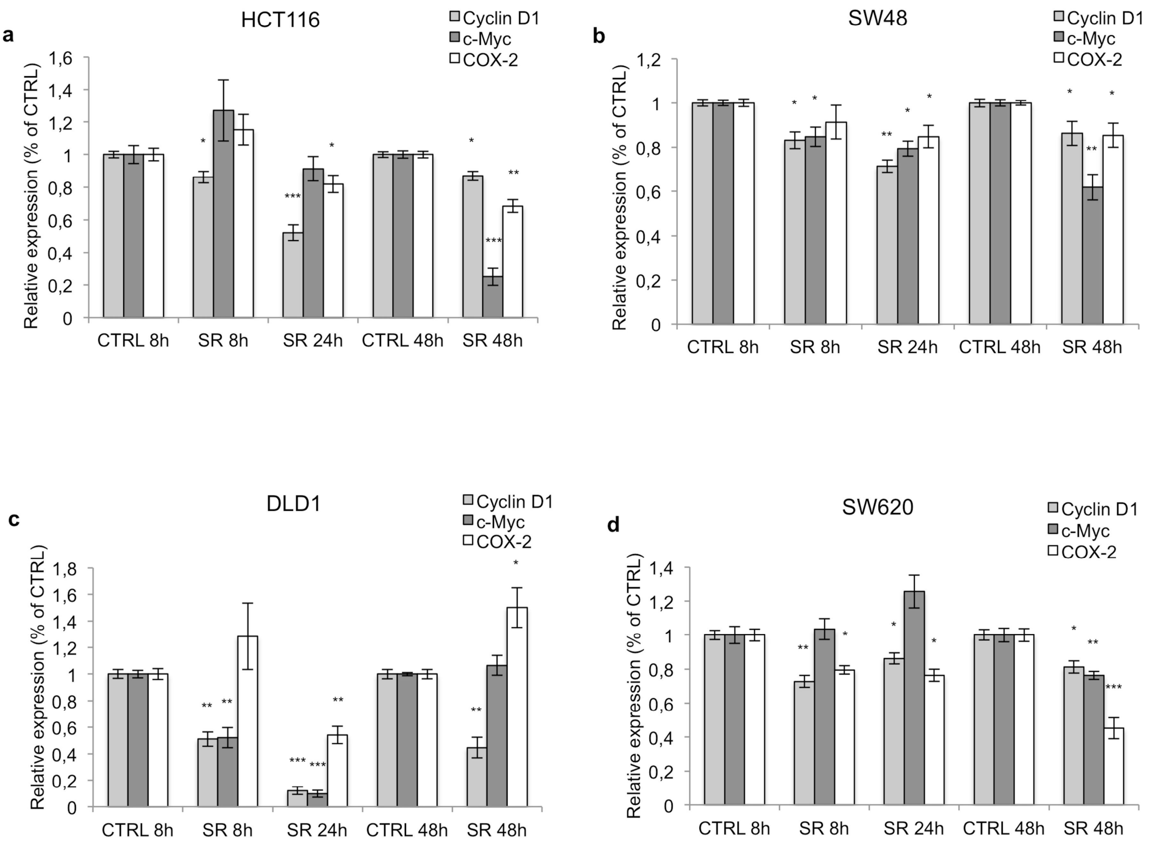

Supplementary Figure S5

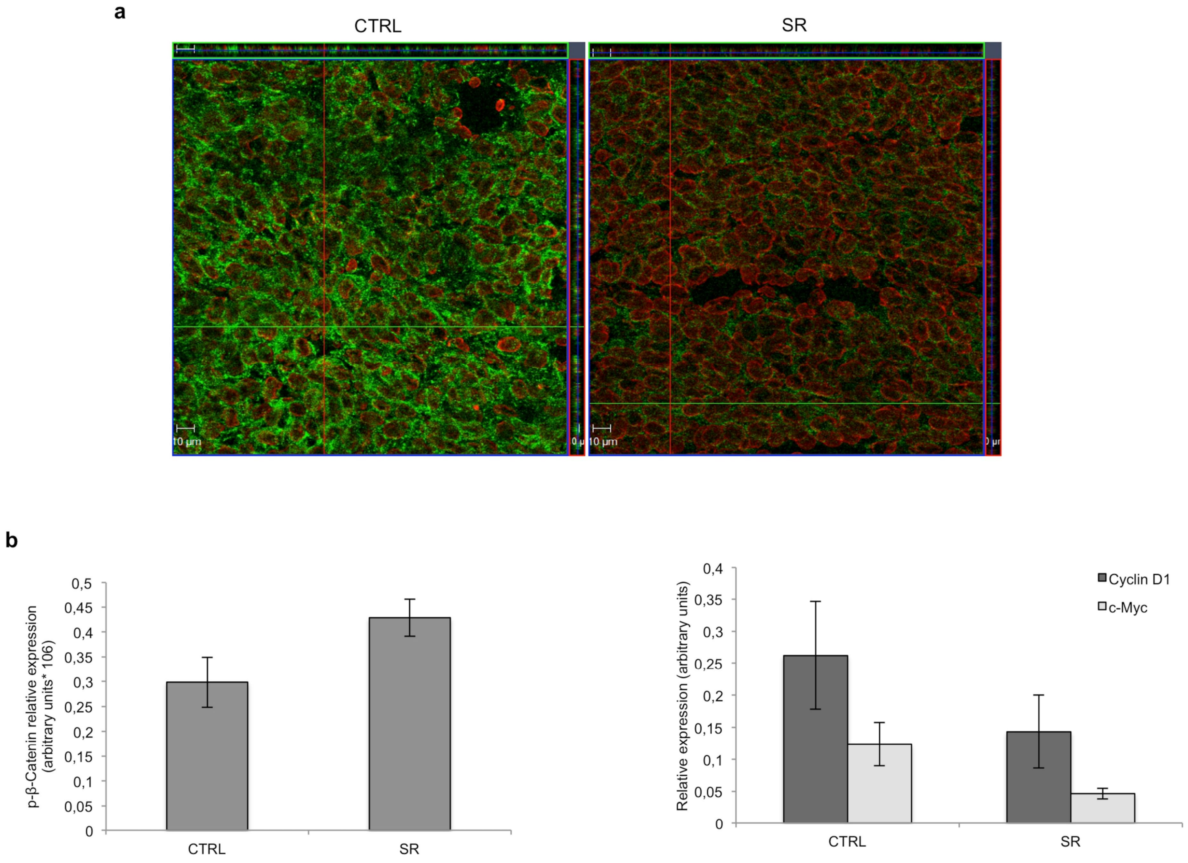

Supplementary Figure S6

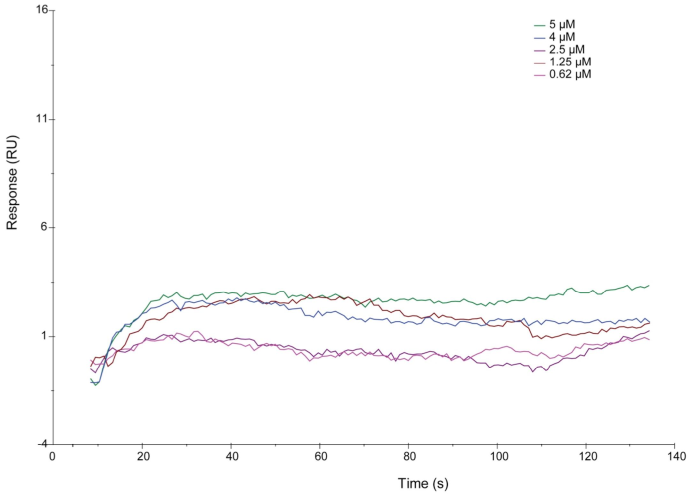

Supplementary Figure S7

Figure 1 d

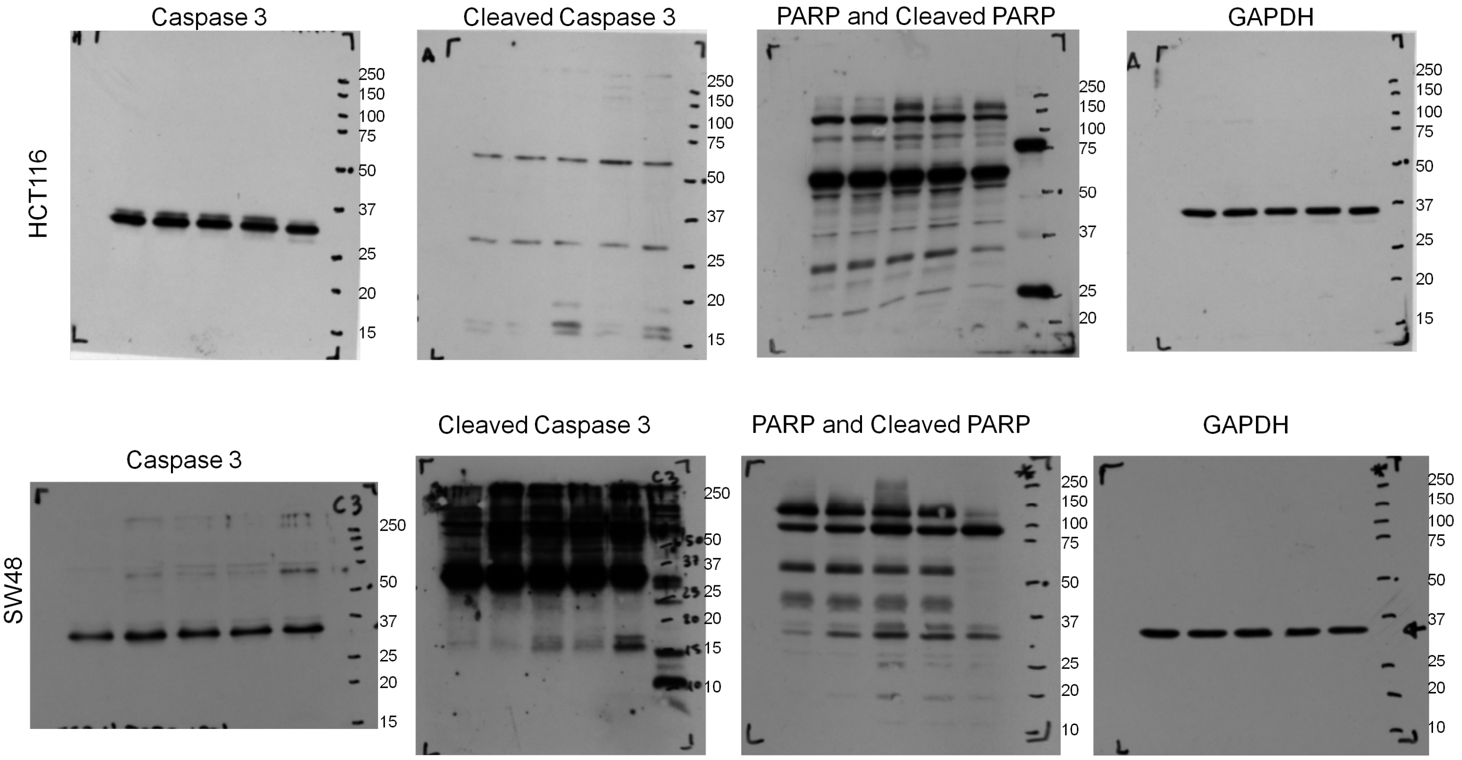

Supplementary Figure S8

Figure 2

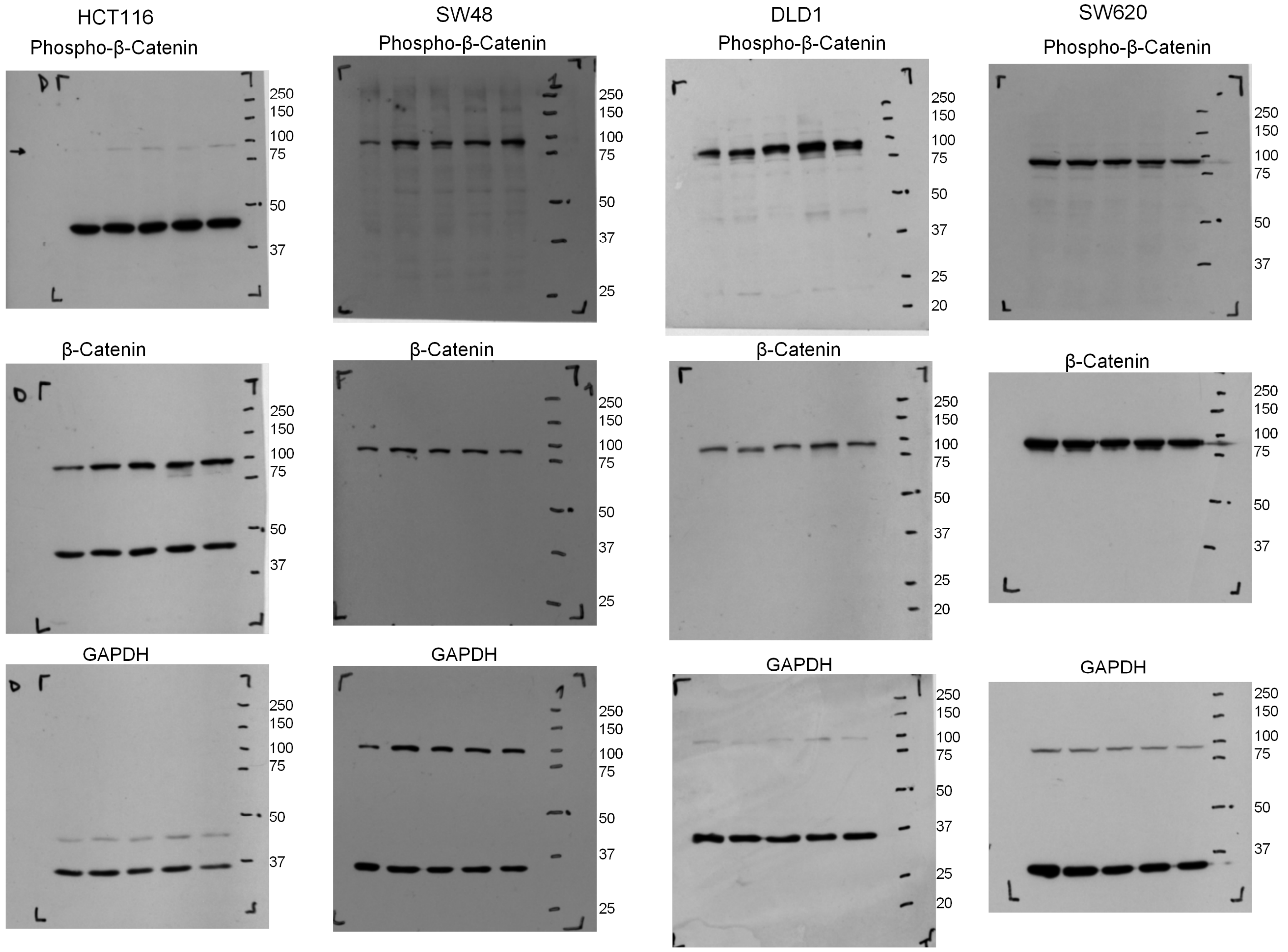

Supplementary Figure S9

Figure 3 a

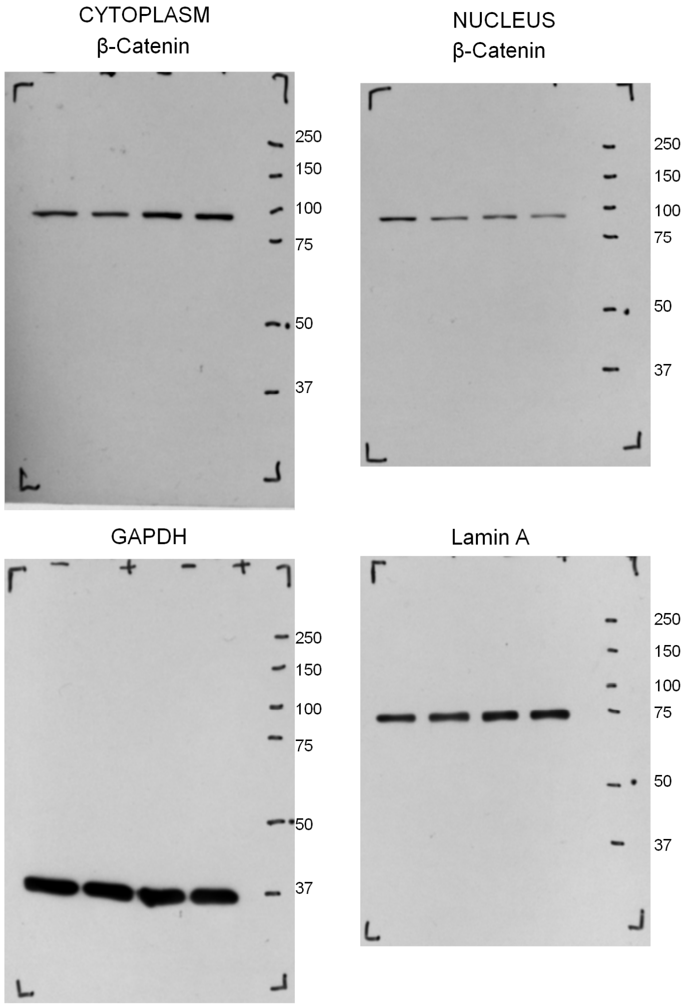

Figure 3 b

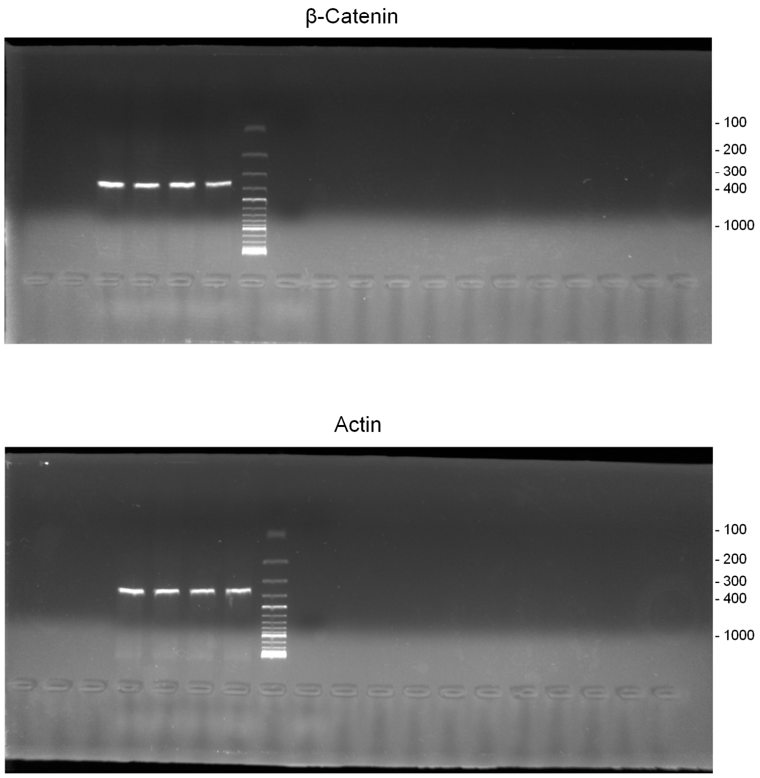

Supplementary Figure S10

Figure 4 a (HCT116, left panel)

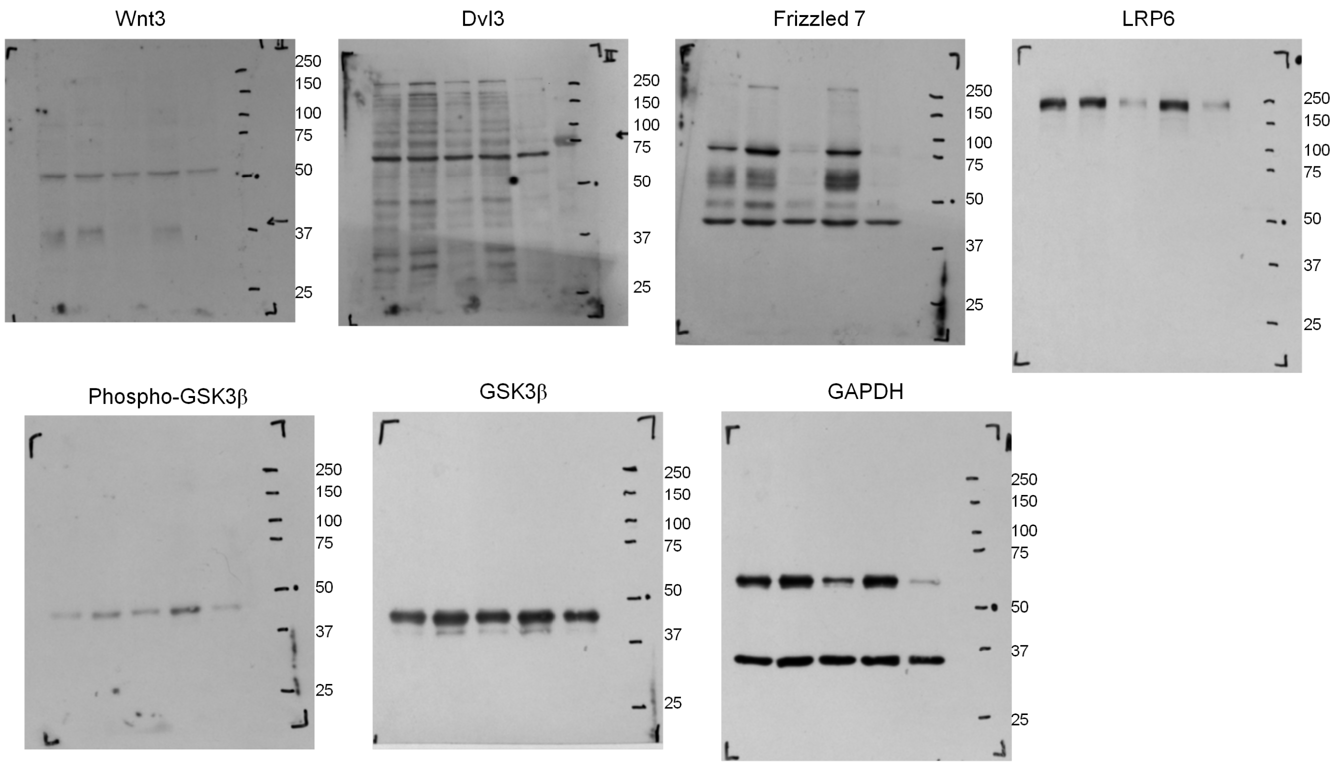

Supplementary Figure S11

Figure 4 a (SW48, right panel)

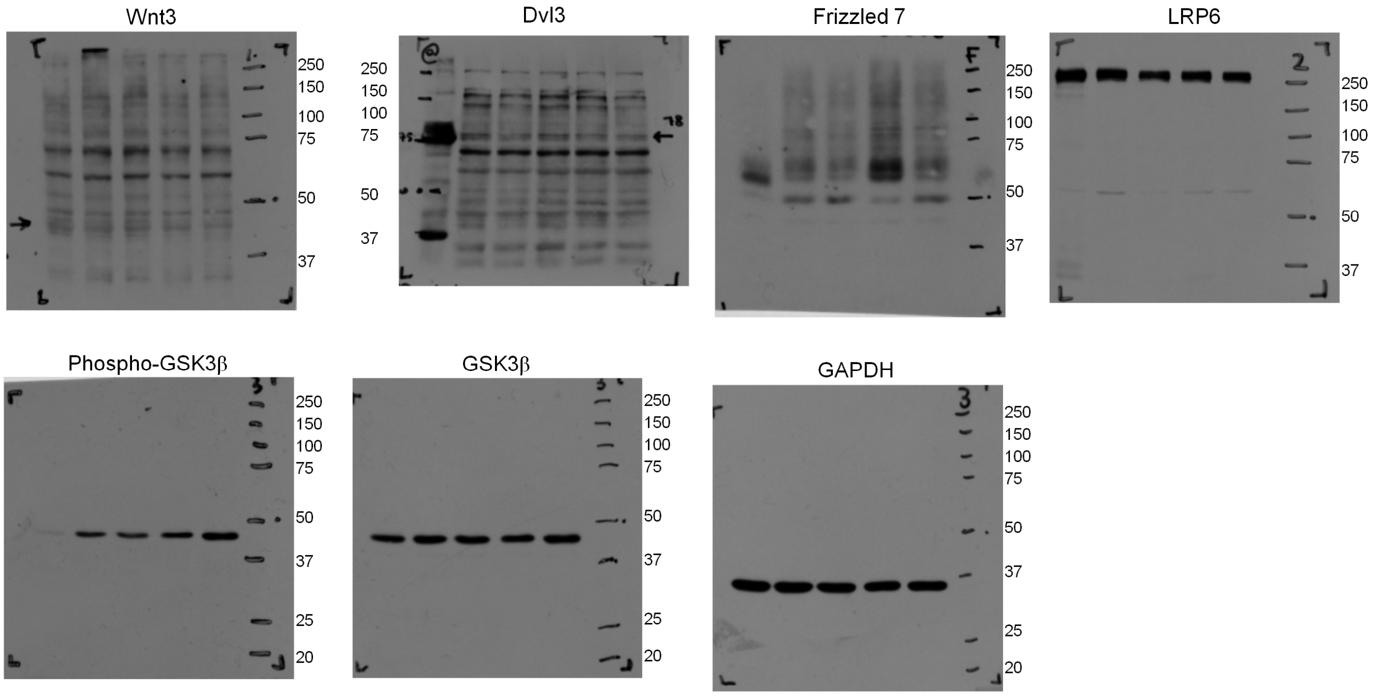

Supplementary Figure S12

Figure 4 b (HCT116, upper)

APC

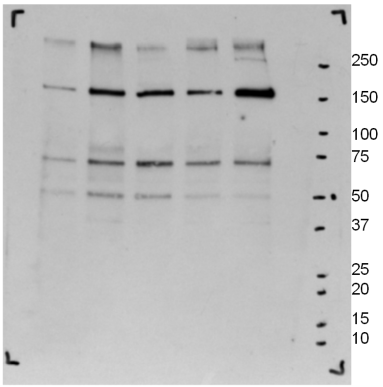

GAPDH

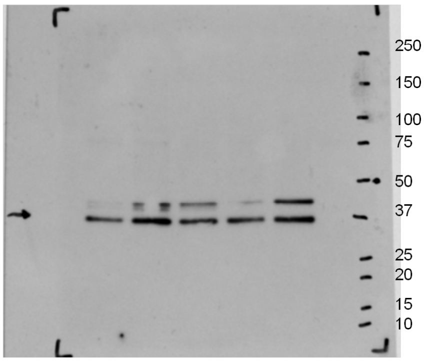

Figure 4 b (SW48, lower)

APC

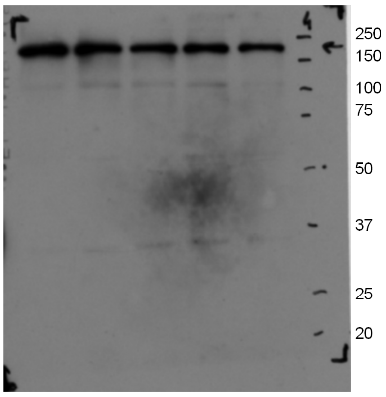

GAPDH

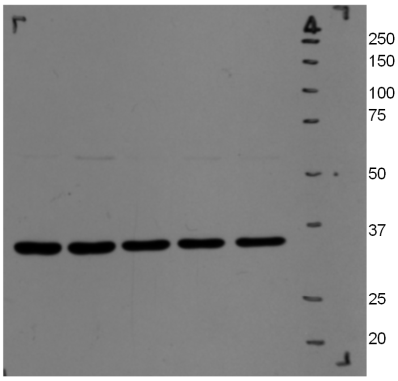

Supplementary Figure S13

Figure 4 c (HCT116, left panel)

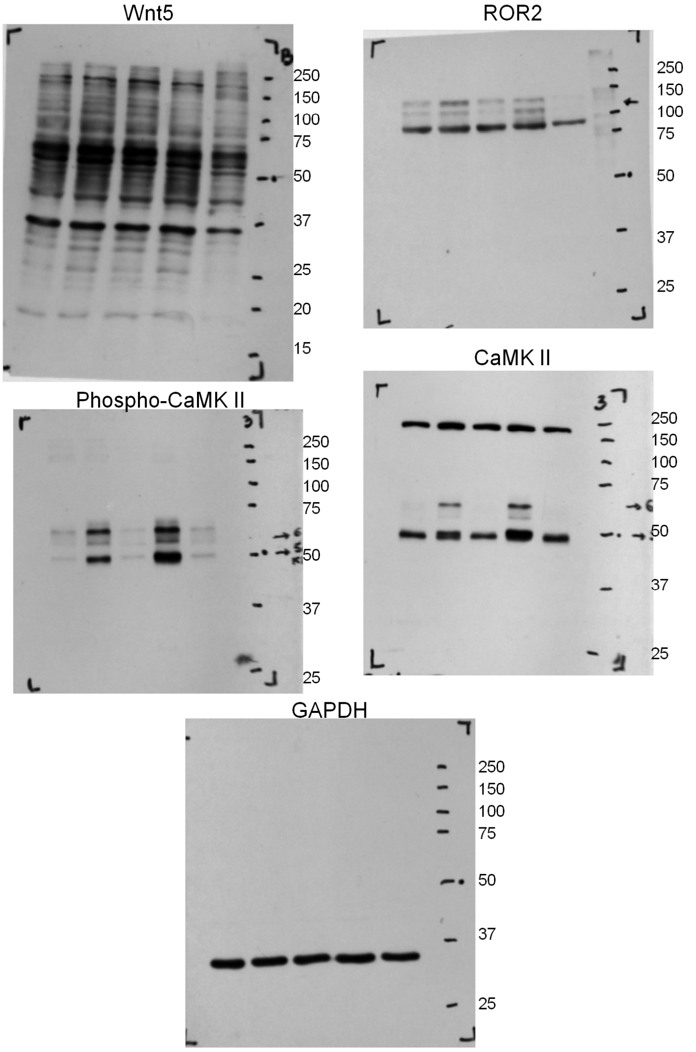

Figure 4 c (SW48, right panel)

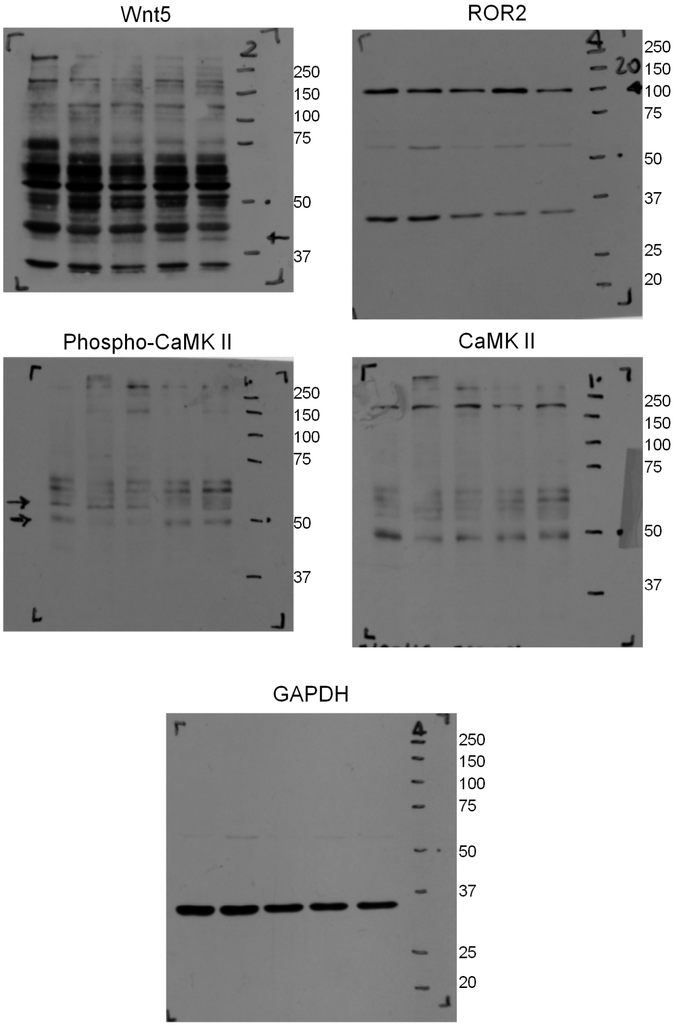

Supplementary Figure S14

Figure 5 b

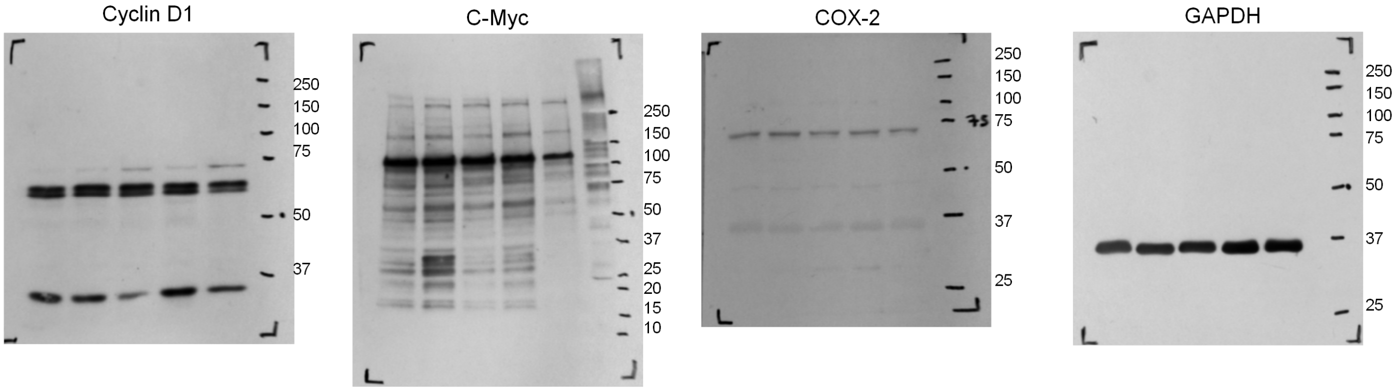

Figure 5 c (SW48)

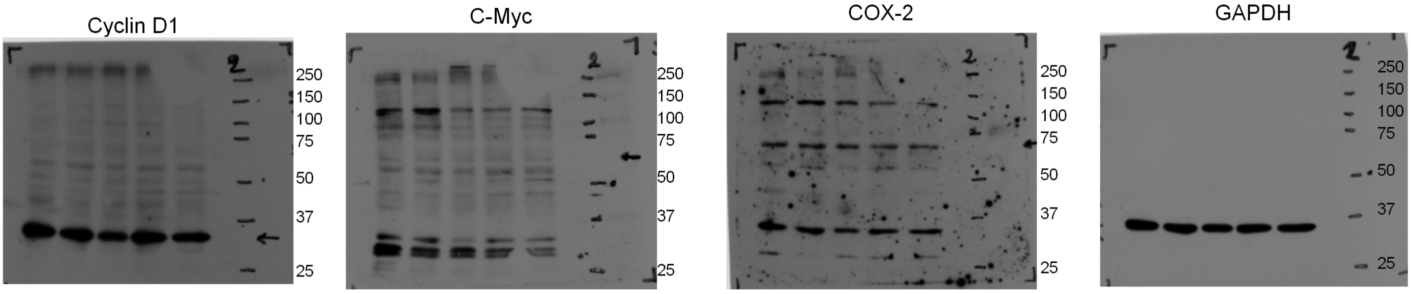

Supplementary Figure S15

Figure 5 c (DLD1)

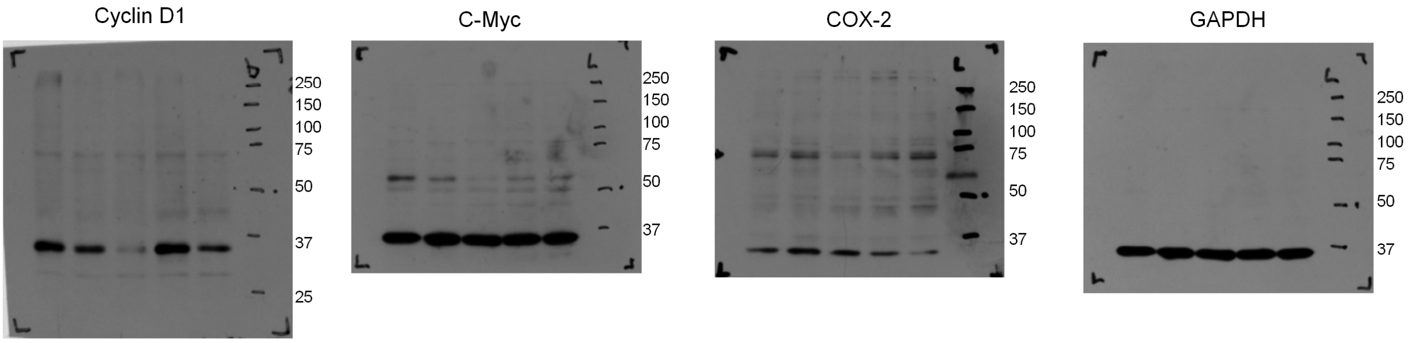

Figure 5 c (SW620)

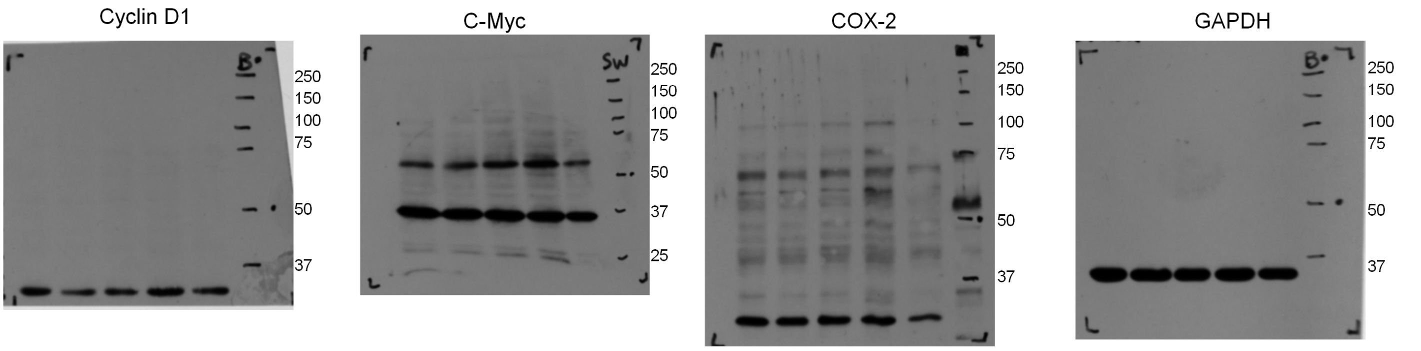

Supplementary Figure S16

Figure 6 d

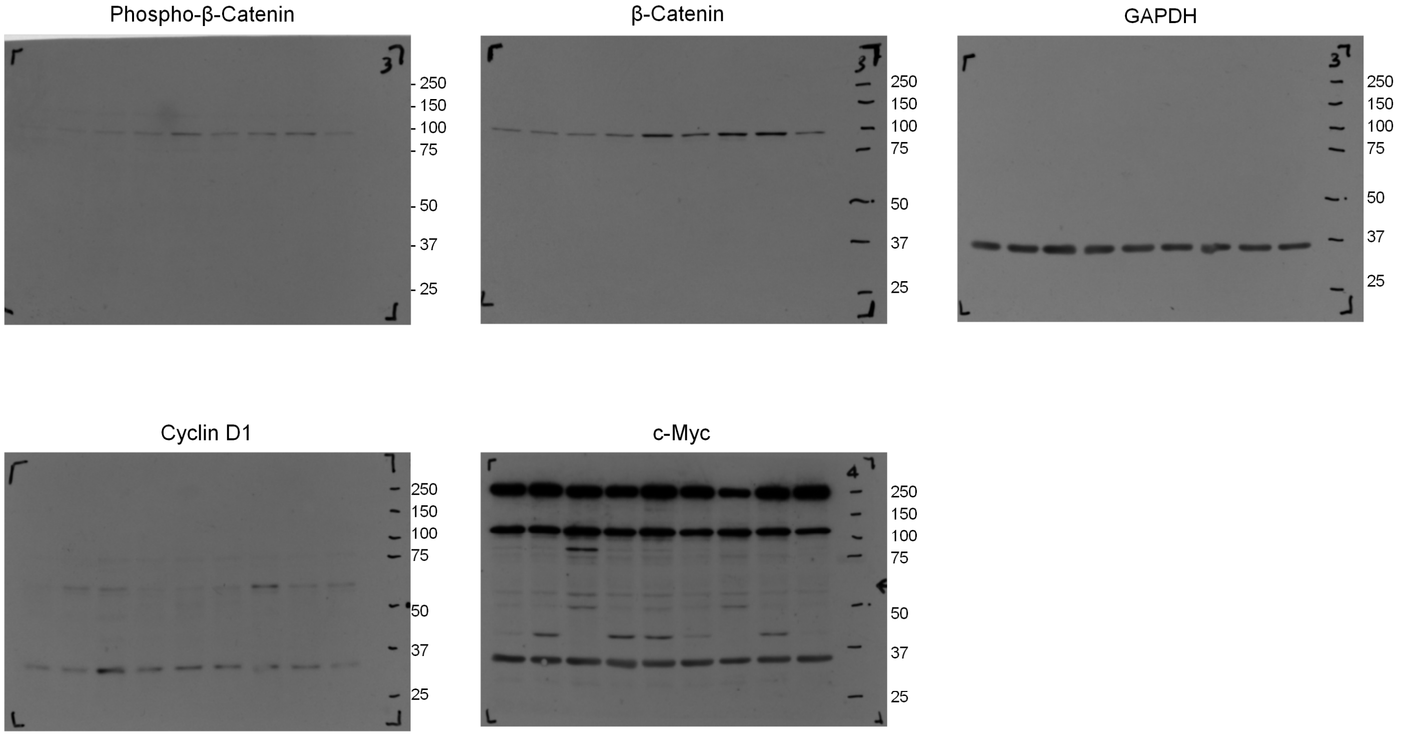

Supplementary Figure S17

Figure 8

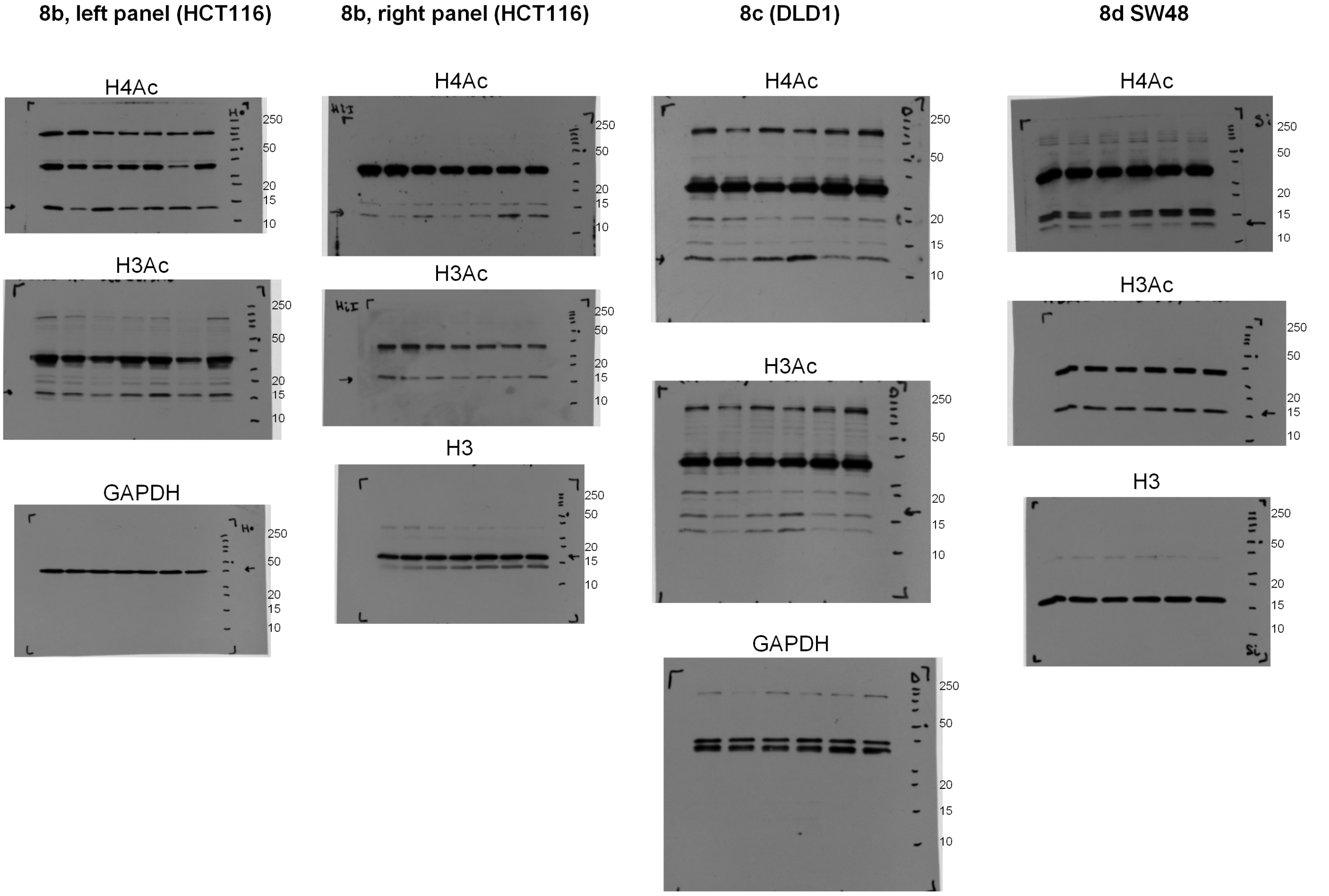

Supplementary Figure S18

Supplementary Figure 4 b

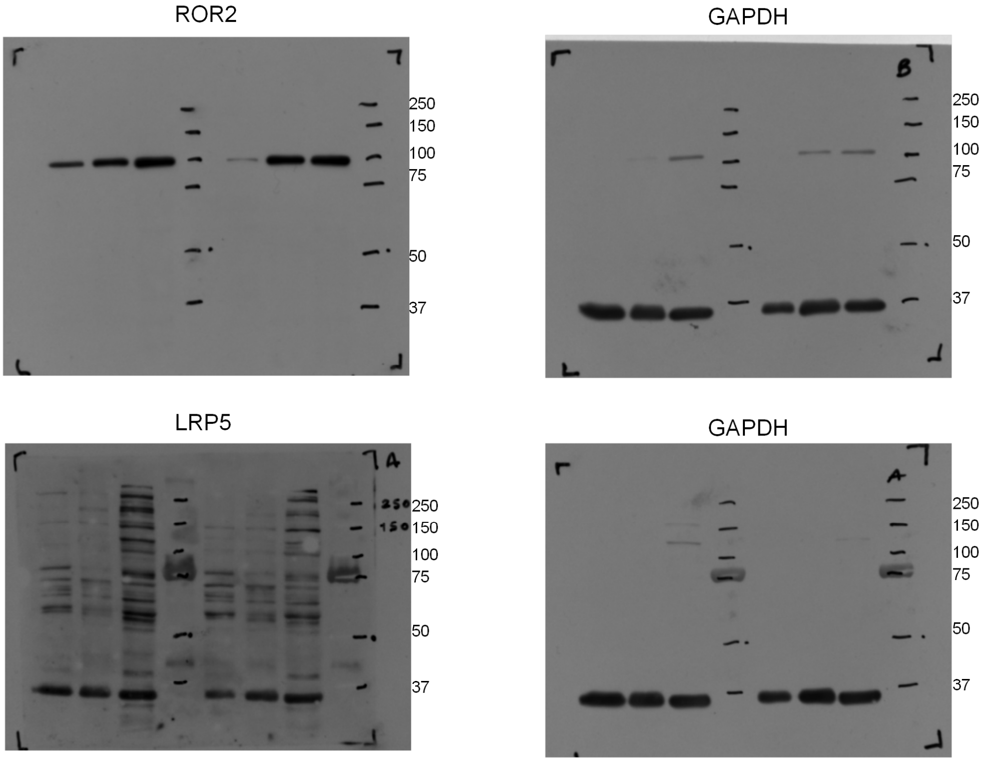

Supplementary Figure S19
